# Aircraft emissions of ultrafine particles characterized by real-world near runway measurements

**DOI:** 10.1038/s41612-025-01095-9

**Published:** 2025-06-19

**Authors:** Jeff Maes, Spyros Bezantakos, Luccas K. Kavabata, George Biskos, Irene C. Dedoussi

**Affiliations:** 1 Operations & Environment Section, Faculty of Aerospace Engineering, Delft University of Technology, Delft, the Netherlands; 2 Climate and Atmosphere Research Center, The Cyprus Institute, Nicosia, Cyprus; 3 Faculty of Civil Engineering and Geosciences, Delft University of Technology, Delft, the Netherlands; 4 Whittle Laboratory, Department of Engineering, University of Cambridge, Cambridge, UK

**Keywords:** Atmospheric chemistry, Environmental monitoring, Engineering, Environmental impact

## Abstract

Aircraft emissions of (ultra)fine particles during landing and take-off operations pose increasing human health hazards for airport employees and near-airport communities. Measurements of in-operation aircraft are therefore crucial for characterizing real-world aircraft emissions, and their variability. In this work, we develop an approach that enables the gathering of large quantities of data on real-world aircraft-specific emissions. We use three types of portable PM sensors located ca. 200 m downwind of an operational runway at Amsterdam Airport Schiphol, over different seasons, to characterize the plumes from ca. 500 specific operations covering most aircraft types of the global flying fleet. High concentration peaks (in the order of 10^6^ particles/cm^3^) of sub-25 nm particles are observed in the near field. While departure plumes exhibit higher particle number concentrations than arrival plumes, the values do not necessarily scale with aircraft size or engine thrust rating. We find large variability among aircraft types and engine models, highlighting the importance of incorporating real-world observations when assessing the impacts of aviation on the atmospheric composition and human health.

## Introduction

Ambient particulate matter (PM), consisting of solid particles or liquid droplets of varying sizes, presents a growing public health concern, with epidemiological studies indicating human exposure to PM pollution as one of the leading causes of premature death globally^1–4^. Among all emissions sources, combustion emissions comprise a key contributor of both fine (i.e., PM_2.5_: particles having sizes 2.5 µm and smaller) and ultra-fine particles (UFPs; i.e., particles having sizes 0.1 µm and smaller). Acknowledging the increasing evidence of the detrimental importance of different PM sizes^5,6^, ongoing efforts aim to better characterize individual contributing sources^7–9^.

Aviation is a growing source of PM with a unique profile of emissions. Annual aircraft fuel consumption in 2019 was 55% higher than in 2005^10^, while the sector has an anticipated growth of 2.4–3.8% annually through mid-century^11,12^. This is expected to continue to further increase fuel consumption and corresponding emissions, unless significant technological or market-based measures are introduced. Directly emitted PM forms a small part of aviation-related PM_2.5_ and associated human health impacts when expressed in terms of mass, but that does not hold when expressing PM emissions in terms of number of particles^13,14^. In 2019, the most recently representative year for aviation prior to the COVID-19 pandemic, aircraft are estimated to have emitted 9.68 Gg of non-volatile PM (nvPM) and ca. 3.47 × 10^26^ particles^10^. It is estimated that 21.3% of the emitted nvPM mass and 11.8% of the emitted nvPM numbers is associated with the landing, taxi, and take-off (LTO) cycle of flight, within ca. 900 m (i.e., 3000 ft) from the surface, disproportionately affecting the vicinities of airports compared to other areas^15^. Besides the air quality and human health impacts, nvPM emitted by aircraft, especially at higher altitudes, also affects the climate by altering the properties of condensation trails (contrails), and through other pathways, including aerosol-radiation and aerosol-cloud interactions^16^.

International mass- and number-based nvPM emissions standards (ICAO CAEP/11) are applicable to new and in-production aircraft engines with rated thrust greater than 26.7 kN from 1 January 2023^17^. As part of this regulatory effort, aircraft engine manufacturers determine and report the nvPM emissions performance of the engines using static tests, which are primarily part of the engine certification process^18^. These data, together with corresponding gas emissions data, form the basis of all aviation emissions inventories, which are used as inputs to air pollution and climate models for assessing their impacts. However, emissions data obtained from static engine tests during certification may not be representative of real world operations as there are operational, meteorological, as well as other aspects that are not captured in the certification process^19^ and thus not taken into account when assessing the environmental impacts of aviation. These include for example the on-wing performance of the engine (as opposed to the static tests), the operational aging of the engine, the exact fuel used (including its aromatic and sulfur content), the aircraft and engine performance (some operational parameters are at the discretion of the pilot), as well as the varying meteorological conditions. For the gaseous emissions, reports in the literature estimate a ca. 24–30% uncertainty range^20,21^. While no comprehensive estimation of the uncertainty has been performed for nvPM certification emissions indices (EI), recent studies indicate high variability, noting even order of magnitude differences among engine models for the same aircraft type, and that different modeling assumptions can lead to a +120% difference in global annual nvPM mass and −23% difference in number totals^10,22^. Additionally, the volatile PM (vPM) also evolves and contributes to the total PM from aircraft as the hot aircraft exhaust mixes with the ambient atmosphere, cools, and is advected.

Measurements of in-operation aircraft are important for quantifying real-world aircraft (nv)PM emissions and the associated uncertainties. Observations performed in the vicinity of airports provide evidence of the dominant UFP sources that airports represent, but do not enable aircraft-specific analyses to quantify the variability between aircraft types and operations^23–26^. Static engine test-cell measurements at the engine exit plane provide a way of independently comparing with the certification data, but lack the operational considerations^27–29^. Near-runway (less than 500 m) measurements of individual aircraft plumes can provide information on the real-world composition of aircraft emissions^21,30–36^. However, such measurements are typically challenging as they require placing large, power-requiring instruments in proximity of restricted areas near runways, thus resulting in a limited individual aircraft types being measured.

In this work, we develop an approach that utilizes recent advancements in portable PM sensors that provide the flexibility and sampling rate to allow characterization of plumes from individual aircraft operations. We carry out multiple measurements over a period of five days, at a distance of ca. 200 m downwind an operational runway at Amsterdam Airport Schiphol and characterize the plume of ca. 500 individual aircraft operations, over different seasons. To the best of our knowledge, this work provides the first comprehensive PM emissions characterization measurements capturing mass, number, and size of particles emitted by in-operation aircraft, representative of the present-day global aircraft fleet using portable (cost-effective) PM sensors. Overall, this approach enables the gathering of large quantities of data on real-world aircraft-specific emissions, that could be critical for a) better understanding and quantifying the real-world engine emissions of UFP and their variability, b) characterizing plume dynamics, and c) evaluating the impacts of operational and other mitigation efforts (e.g. the increasing blending of Sustainable Aviation Fuels). This additional information can complement established and future long-term efforts to determine ambient air quality in and around airports using higher fidelity (and cost) monitoring equipment based on approved technical specifications that currently form the basis of regulatory datasets.

## Results

### Observed timeseries

**Fig. 1:**
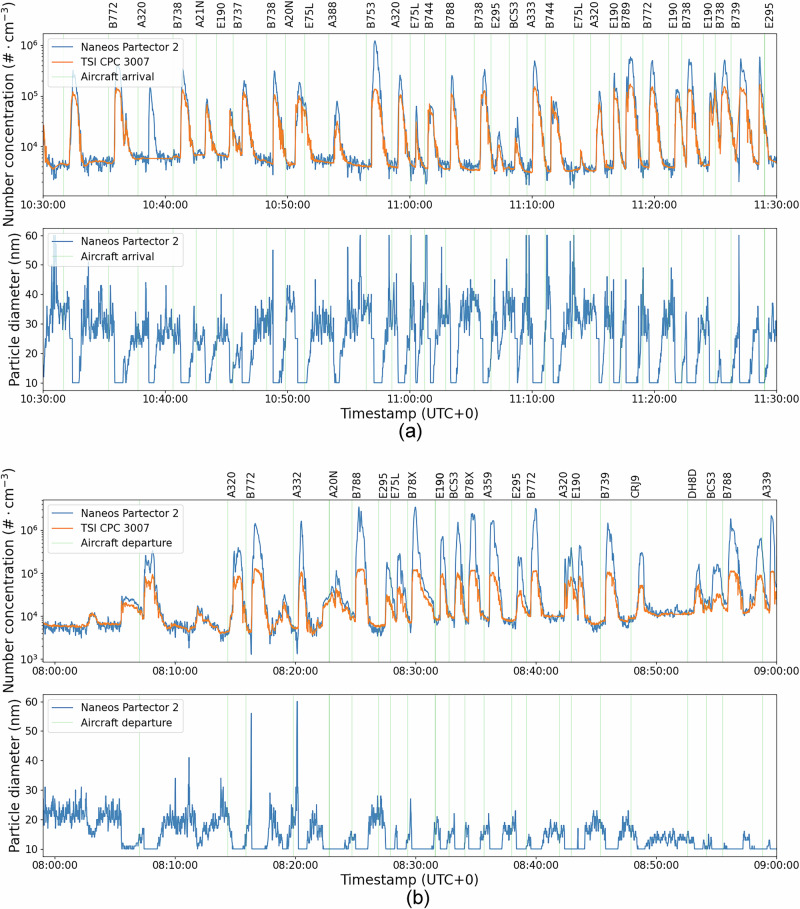
Example timeseries of measured particle number concentration and diameter. Displaying measurements over a 1-hour period showing arrivals (**a**) and departures (**b**), as recorded by the Partector 2 and the CPC (both undiluted in this case). Top row includes the particle number concentration (in #⋅cm^−3^) in logarithmic scale and the bottom row includes the average particle diameter (in nm) derived from the Partector 2. The green vertical lines show the aircraft type departing or arriving.

Three types of portable PM sensors are deployed to characterize the particle concentration and size of the aircraft plumes: a multi-metric nanoparticle detector (Naneos Particle Solutions GmbH, Switzerland, mods. Partector 2 and Partector 2 Pro), a portable condensation particle counter (CPC, TSI Inc., USA, mod. 3007^37,38^), and a custom-made PM monitor that employs a low-cost Optical Particle Counter (OPC)^39^. A diluter was used on some of the measurement days as the measured concentrations exceeded the upper instrument threshold. Figure 1 provides an example of recorded measurements during a number of landings and a take-offs. We observe that each individual aircraft operation is associated with an increase in the measured particle number concentration (referred to as ‘plume’ hereafter), which aligns well with the expected time of observation given the cross-wind speed, having a peak that occurs on average 1.25 minutes following the aircraft. Each plume is also associated with a distinct decrease in the average diameter of particles having diameters down to 10 nm, as measured by the Partector 2 in nearly all cases. We note that 10 nm is the minimum average particle diameter reported by the Partector 2, which does not exclude the presence (and counting) of smaller particles in the plume in significant quantities.

The observed plume characteristics depend on the operational specifics, but some, such as the recorded plume peak, in #⋅cm^−3^, and duration (referred to as ‘width’ hereafter), in s, also depend on the local weather conditions. Figure S3 in the SI shows an example of how these vary among different plume measurements of the B738, which is the type for which we have the most observations. Higher cross-winds are associated with larger peaks and narrower plumes. Since the measurements have been performed over different days, we choose the area under the recorded plume (referred to as ‘plume area’ hereafter), in #⋅cm^−3^⋅s, as the meaningful metric for quantifying the plumes, which is a more robust quantity across cross-wind speeds (as also confirmed by the simulation results presented in the following subsections). Overall, we observe larger peak concentrations, as well as plume areas, for the departures, indicating higher particle number emission rates, which is in agreement with other observations^35^.

The OPC measurements display the largest variations in the PM_2.5_ signal for the smallest bin size (300–550 nm), which may be related to the individual aircraft contributions (Fig. S1 and S2 in the SI). However, since the signals are not consistently present and not multiple times larger than the background (as is the case with the Partector 2 and the CPC measurements), it becomes challenging to confidently match peaks to individual aircraft and extract useful information. Considering that, the OPC results are not discussed further in the following sections.

### Aircraft-specific contributions

**Fig. 2:**
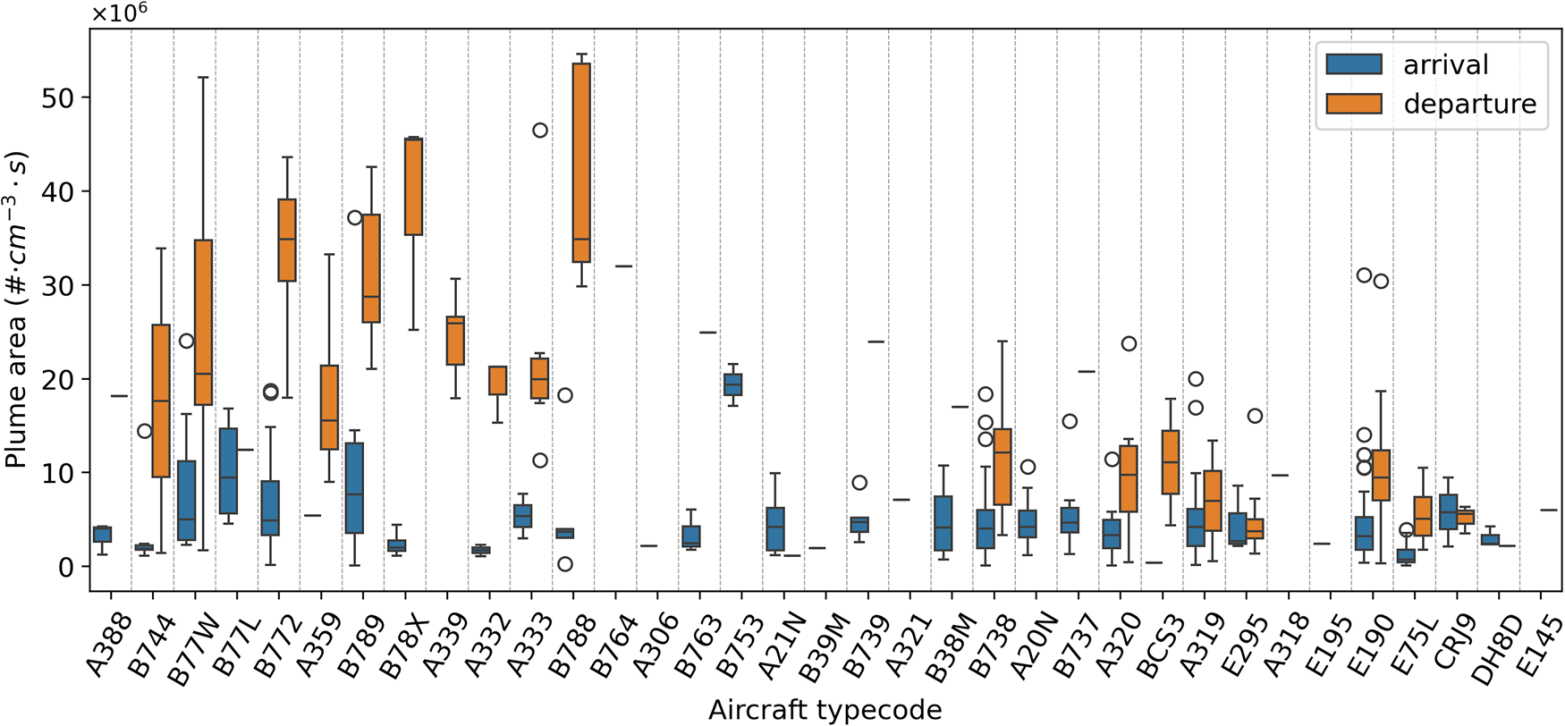
Particle number emissions performance for different aircraft types sorted by decreasing Maximum Take-Off Weight (MTWO), from larger (left) to smaller (right) aircraft. Partector 2 measured plume area (in #⋅cm^−3^⋅s) is shown for both departures and arrivals. Figure S9 in the SI presents diluted and undiluted measurements separately.

Figure 2 presents the measured plume number concentration area for individual aircraft types, ordered by decreasing Maximum Take-Off Weight (MTOW), for probed plumes corresponding to both departure and arrival operations. Overall, for the same aircraft type, departure plumes have larger plume areas, indicative of higher emissions rates. We note however that arrival operations last longer than departures. The emitted number of particles in the plumes does not necessarily scale with the size of the aircraft for neither departures nor arrivals. For example, the E190 (ca. 100 passengers) leads to similar plume areas as the A319 and A320 (ca. 140–160 passengers). However, the difference in the emission rates between single and twin-isle aircraft is larger during departures. While we account for the variability in wind speed and direction between the different observations by using the plume area metric, any remaining variability in plume area for each aircraft type can be related to operational variations, as well as measurement uncertainties, as discussed in the following sections.

**Fig. 3:**
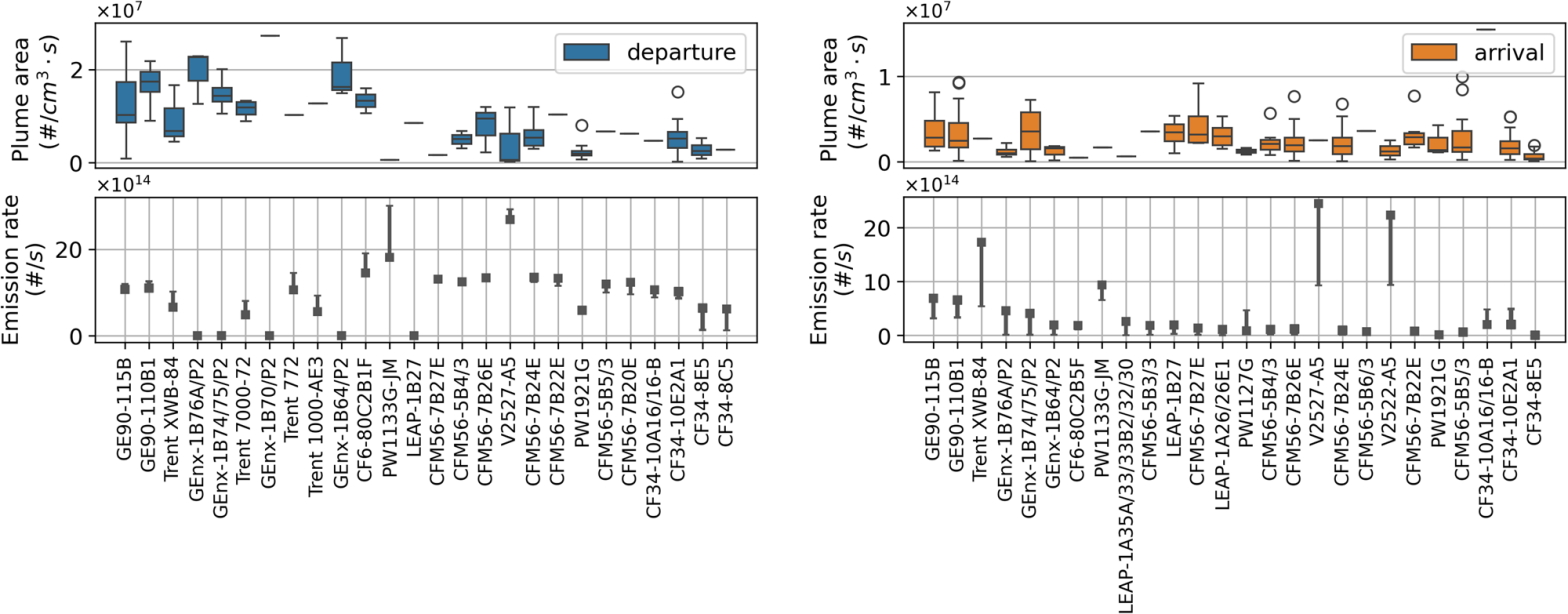
Particle number emissions for different engine models during departure (left) and arrival (right) operations, sorted by decreasing maximum rated thrust. The top figures display the plume area (in #⋅cm^−3^⋅s) determined by the Partector 2 measurements for aircraft powered by each engine, scaled by the number of engines. The bottom figures display the particle number emission rate ranges (in #/s) based on the certification data for relevant thrust settings. For departures, these are the Take-off (100%, shown in square) and Climb-out settings (85%, shown in dash), whereas for arrivals, these are the Approach (30%, shown in square) and Idle (7%, shown in dash) operations. Figure S10 in the SI presents diluted and undiluted arrival measurements separately.

The certification data provided in the ICAO Engine Emissions Databank specify the nvPM emissions index (mass or number of emitted particles per mass of fuel burnt) for specific thrust settings, which, together with the reported fuel flow, can be used to calculate the expected nvPM particle number emissions rates (in #/s). Direct comparison to these nvPM certification data with the measurements provided here is challenging, as we do not separate the nvPM component from the total PM measured, and we also lack information on the exact thrust setting or the exact fuel flow of each aircraft. However, it could be expected that a higher nvPM emission rate (#/s) would correlate with a larger observed plume area, thus allowing a relative comparison between expected nvPM emissions rates and measured total PM plume area. This relative comparison is presented in Fig. 3, which shows the measured plume area per engine (top row figures), as well as the reported nvPM emissions rate for two likely thrust settings during arrivals and departures (bottom row figures). For departures, we observe a relationship between the rated thrust and resulting emissions, while this is not evident in the certification data. In addition, the measurements do not identify the ‘high emitters’ (e.g., V2527-A5) reported in the certification data. However, we note that this relative comparison is limited by the ratio of volatile to non-volatile PM present over different meteorological conditions. Attempts to understand directly from the measured data of the B738 the dependence of the plume area on the temperature (ranging from 3 °C to 21 °C) and relative humidity (ranging from 42% to 100%), that could give an indication of the variability of the vPM component of the total PM measured, did not yield clear conclusions (Fig. S4 in the SI). Additionally, we note that operational choices, such as the use of reverse thrust, and the assumed aircraft-engine combination, could affect this comparison.

**Fig. 4:**
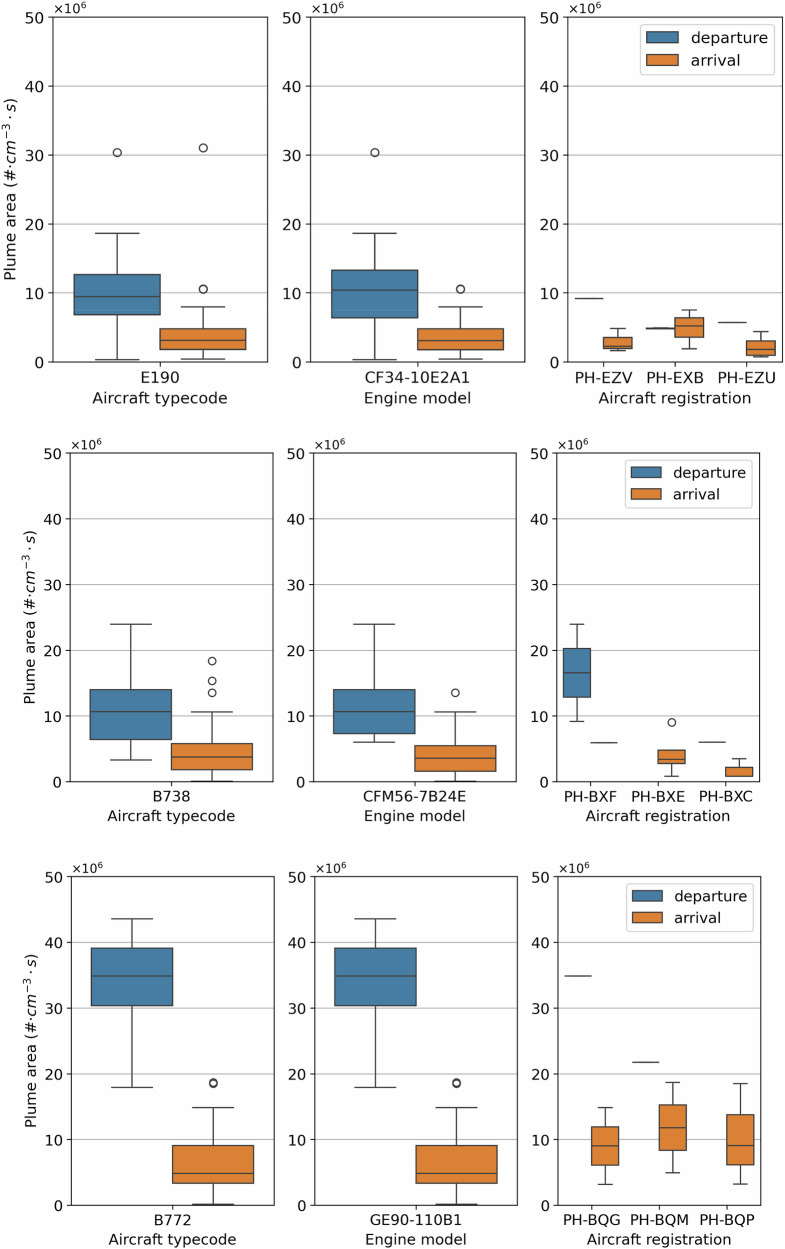
Measurement variability for commonly observed aircraft types and registrations. Plume areas (in #⋅cm^−3^⋅s), determined using Partector 2 measurements, are provided for three different aircraft types: an Embraer 190 (top), a Boeing 737-800 (middle), and a Boeing 777-200 (bottom). Left plots show all observations for each of the three aircraft types, center plots show all observations for a specific engine model that powers each of the three aircraft types, and right plots show all the observations for three individual aircraft registrations of that aircraft type and engine model. The number of observations decreases from left to right.

The measurement variability observed between the same aircraft type, as shown in Fig. 2, can be the result of operational and meteorological variations, as well as measurement uncertainties. Figure 4 expands on the observed variability in the plume area within the three commonly observed aircraft types of different sizes (E190, B738, and B772), eliminating parameters that affect the observed plumes, one at a time, from left to right, at the expense of the statistical sample size. The left column of the figure provides data from all observations for that aircraft type, the middle column for one engine model (the one that powers the registrations shown), and the right column for three commonly observed aircraft registrations (tail numbers) within the multiple measurement days. With the exception of the largest aircraft (B772), there is a reduction in measurement variability from the aircraft type (left column) to the specific registration (right column), as expected. This indicates that our measurements are not dominated by instrument uncertainties, but are representative of the real-world emissions variability. For the B772 aircraft, the variability does not change substantially. The reason for this is in part that only one engine model was assessed, while the measurements for the same registrations did not necessarily take place on the same day. As a result, the remaining variability could be indicative of the impact of different operational and meteorological conditions or the emissions performance degradation over time. The same is true also for the B738 and the E190 aircraft. Overall, we note that the number of measured samples for the individual registrations is 3–4 for arrivals, and that more extensive measurements could strengthen this comparison.

Nucleation mode particles (i.e., particles smaller than 25 nm) are primarily observed in both arrivals and departures. In terms of particle size, as determined by Partector 2 which estimates the average particle diameter, we observe a smaller average particle size for departure plumes than for arrival plumes. The size distribution of plumes from 176 arrival operations is analyzed using the Partector 2 Pro and summarized in Fig. S13 in the SI, for different aircraft types. The plumes, in their majority (in terms of number concentrations), include particles residing in the two smallest bins (10.0 nm and 16.3 nm). Finally, we do not observe significant differences when analyzing the results per engine model.

### Drivers of the variability in the near field measured aircraft PM

By analyzing ca. 500 plumes over different seasons from aircraft types representative of the global fleet, we find significant variability in the plume particle number concentration, even for the same aircraft type. We discuss the three main sources below and note that this real-world particle variability could be better captured by aircraft emissions inventory compilations, especially for studies that focus on fine-scale air quality within and around airports.

#### Meteorological variability

As previously noted in the literature^40^, meteorological conditions can affect the plume particle number concentrations. While the variability is high, we note a trend of lowest plume areas under lower and higher temperatures, however, more observations are necessary to draw a concrete conclusion (SI Fig. S4).

**Fig. 5:**
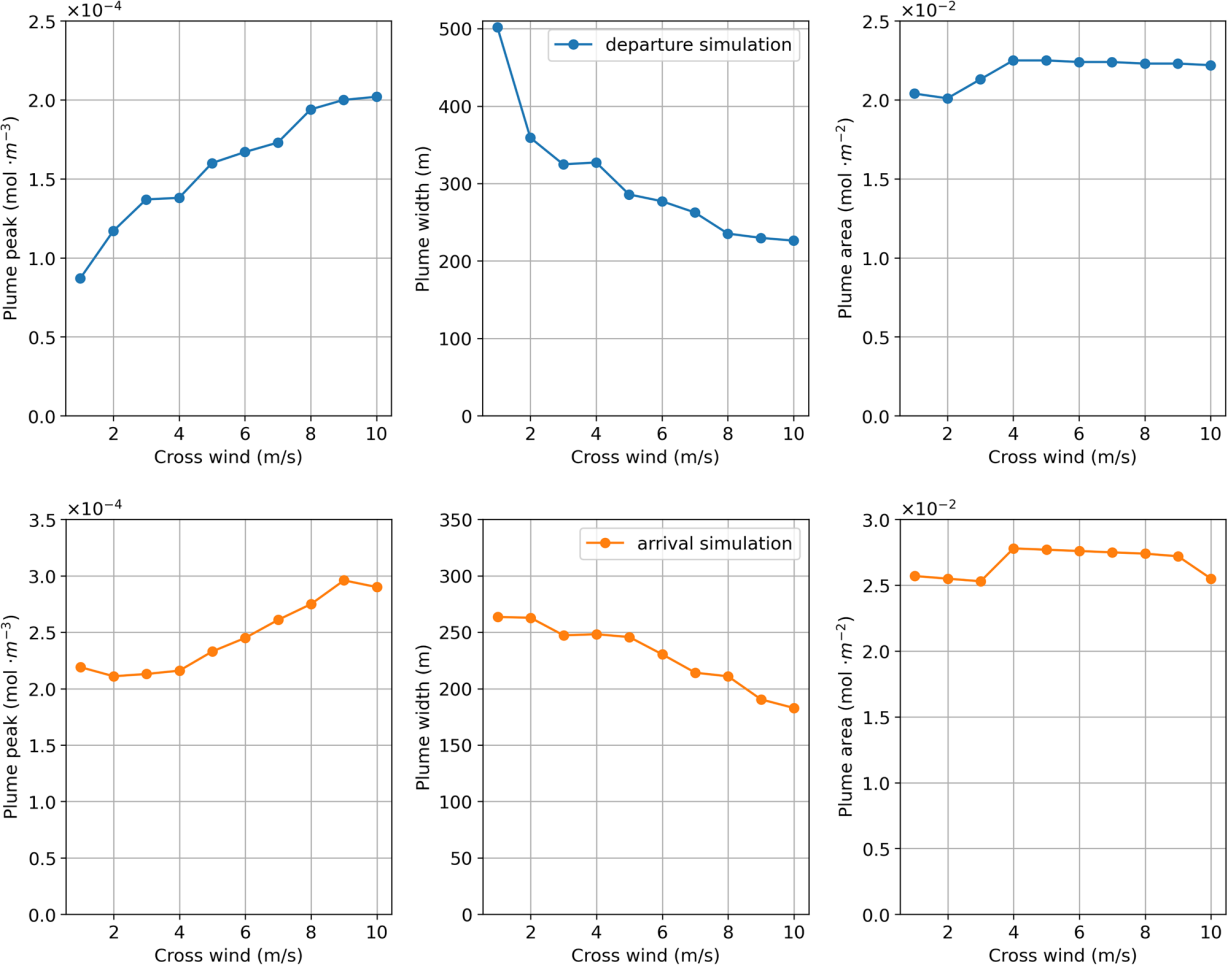
Plume peak in mol/m³ (left), width in m (middle), and area in mol/m² (right) versus crosswind speed. The upper plots (blue) represent the plume properties for a departure configuration, whereas the lower plots (orange) for an arrival configuration.

We use numerical simulations to better understand the expected variability in the measured plume metrics under different wind conditions, and thus measurement days. Figure 5 shows the variability in the plume metrics (peak, width, area) for different crosswind speeds. As expected, we observe higher peaks and narrower plumes for higher cross-winds. The plume area is, however, more robust among different wind conditions, thereby confirming our choice of this metric for the comparison between different measurement days. We also observe this in the measurements (SI Fig. S3), albeit with more noise.

On average, the measured plume width for the B738 aircraft is within ca. 25% of that of the simulated plumes. Specifically, the simulation results show a plume width of 307 m for departures, compared to the average measured plume width of 230 m (standard deviation of 59 m). For arrivals, the simulation results show a plume width of 247 m, compared to the average measured plume width of 183 m (standard deviation of 74 m). Further details about the comparison can be found in Section S3 of the SI.

#### Instrument uncertainty

**Fig. 6:**
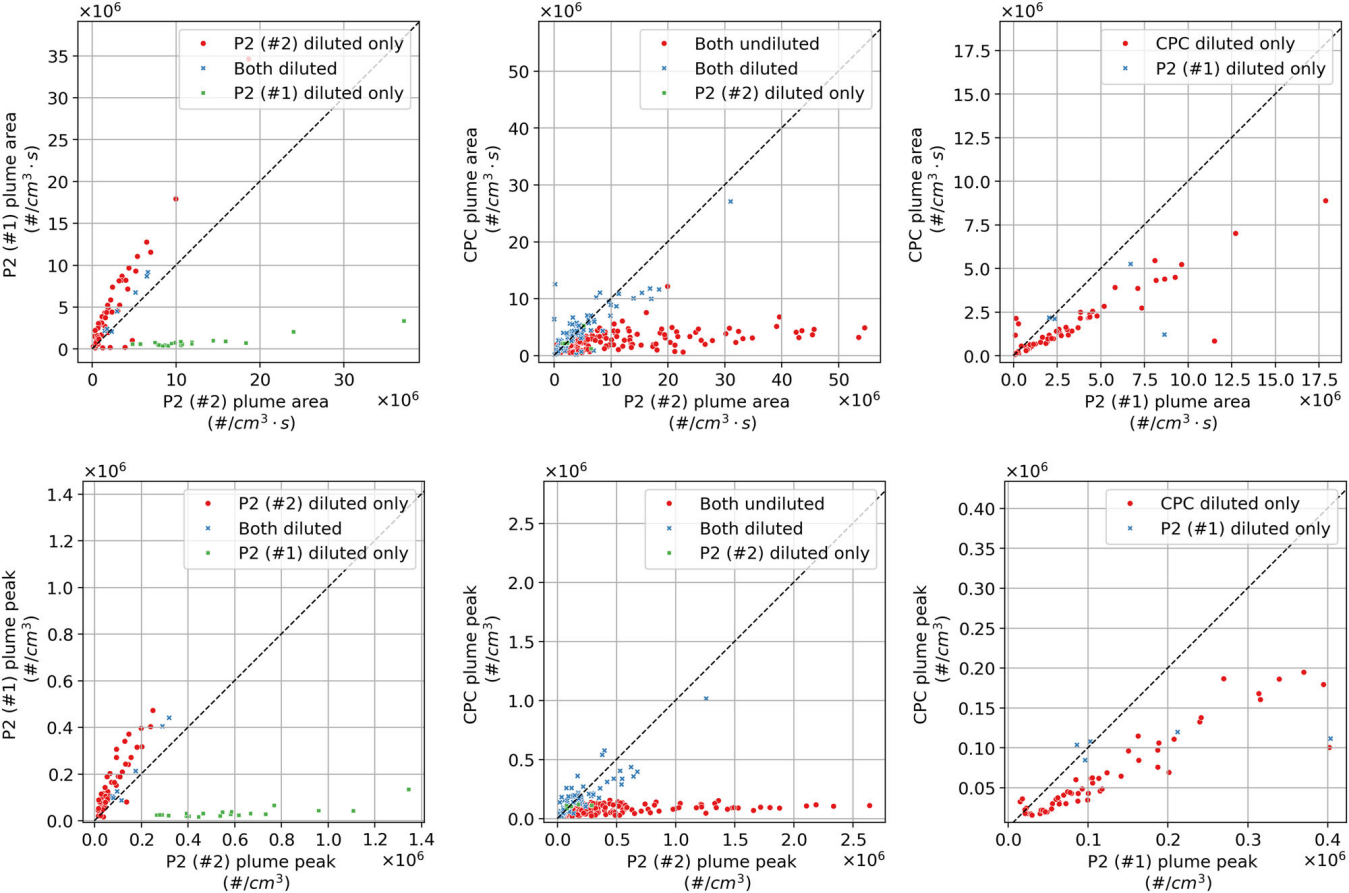
**Instrument comparison.** Plume area (in #⋅cm^−3^⋅s, top row) and plume peak (in #⋅cm^−3^, bottom row) comparison between the two Partector 2 systems and the CPC, for cases when the sample of neither, one, or all the instruments was diluted.

Some of the presented variability is expected to originate from the instruments deployed, given their accuracies (i.e., 30% for the two Partector 2 and 20% for the CPC as reported by the manufacturers, and applicable within the operational envelope of the instruments). Figure 6 compares measurements of the same aircraft plumes captured with multiple instruments, including measurements where the samples were both diluted (and corrected for the dilution ratio) and undiluted. Overall, we observe larger concentrations when the instruments are diluted, even when operating within the upper instrument threshold. We also observe the expected underestimation (in terms of plume area, but not plume width) when using the CPC undiluted, given the relatively low upper instrument particle number concentration threshold (10^5^ particles/cm^3^). Details on parameters that could be driving these effects are provided in SI Section 4.2 in the SI.

## Discussion

We show that portable (and intermediate cost) particle instruments can be used to characterize the real-world ultrafine particle emissions of in-operation aircraft. We perform measurements over multiple seasons, ca. 200 m laterally downwind of an operational runway, capturing the plumes from ca. 500 aircraft from both arrival and departure operations. We use the plume area as the quantity representative of the emissions of each aircraft to account for the different meteorological conditions that would affect the plume dynamics between the engine exit plane and the measurement location. Overall, we find concentrations of sub-25 nm particles in the order of 10^6^ #/cm^3^ in the near field, with substantial variability, even between the same aircraft type.

Our work adds to the existing body of literature on real-world PM measurements from aircraft engines that have been obtained with higher-end particle measuring equipment^21,30–36^. Our results are consistent with observations by Moore et al.^33^, Lobo et al.^31^, Takegawa et al.^34^, and others^36,41^ regarding the small sizes (in some cases <10 nm) of the emitted particles, despite presenting total PM, as opposed to isolating its nvPM component. Our results are consistent with Stacey et al.^35^ who also recorded higher number concentrations for departures than arrivals. As reflected in the review of Stacey (2019)^42^, quantitative comparisons between studies become challenging due to the different assumptions and presentation of the data. Because of the area plume metric used here and the lack of direct or indirect information on the fuel burn, a direct comparison with the emissions indices presented in other works is not possible. However, unlike other works, we partly eliminate the effect of plume dispersion and dynamics when comparing aircraft, by estimating the integrated plume area. Similarly, since the ICAO engine certification data are expressed in terms of nvPM emissions indices for a specific thrust setting, a quantitative comparison with, and direct evaluation of, the certification data has not been possible in this work. This could be partly addressed by expanding the observed species to include concentration measurements of CO_2_, which scales with the fuel burnt, and would thus enable the estimation of the emissions index. The specific thrust setting, however, would need to be either obtained via Flight Data Recorder (FDR) data from individual airlines or could be extracted from aircraft noise measurements^43–45^. Finally, instrumentation for separating the nvPM and vPM components in the total PM measured would also be necessary.

This work was in part motivated by the need to better characterize the real-world variability of particle emissions from aircraft. Examples of parameters likely to affect the near-field PM emissions from aircraft include the thrust setting, exact touchdown and lift-off points along the runway, the weight of the aircraft, the specific engine model it is equipped with, the operational age of the engines and time since the last maintenance cycle, the exact fuel type in the case of arrivals (arriving aircraft likely fueled at the origin airport prior to departure with fuels of different compositions), the wind velocity and related age of the observed plume, the ambient temperature and relative humidity, and potentially other unknown effects. We find, under real-world conditions, significant variations in particle concentrations, even among the same aircraft and engine types. While some of this variability might be driven by non-aircraft factors (e.g., measurement equipment), we find lower variability when measuring the same aircraft registration (i.e., identical aircraft) multiple times.

Out of the instruments employed, the Partector 2 (including its Pro version) and the CPC were the more reliable ones. We were not able to identify clear plumes associated with individual aircraft operations in the data from the custom-made OPC. This is not surprising considering that the particles emitted from individual aircraft operations are in the ultrafine size range, with the majority of them having sizes below 25 nm, thus being undetectable by the OPC. In general, instruments that rely on light-scattering techniques for measuring particle size distributions and number concentrations as well as reporting PM_2.5_ mass concentrations are more likely to underestimate the impact of aircraft/airport operations on local air quality (cf. further information Section S1.4 of the SI). Additionally, the use of a diluter proved necessary as the measured concentrations often exceeded the upper instrument threshold. Even when operating within the instrument threshold, the corrected results indicated higher concentrations than when a diluter was not present. While this does not substantially affect the conclusions drawn here, future work could assess the sensitivity of the dilution ratio on the instrument performance and the reported results, and formally consider potential particle losses in the diluter system, which are currently not accounted for.

The measurement uncertainty could be quantified and reduced, to some extent, by incorporating higher-end equipment, e.g., a Scanning Mobility Particle Sizer (SMPS) as has been done in previous studies^30^. While the response time of such high-end instruments may be much larger compared to the ones required here, in many cases exceeding the plume durations observed in the near field, they can provide highly resolved and highly accurate complementary measurements of particle size distributions, which can help to better understand the real-life aerosol dynamics in aircraft exhaust plumes, especially in the far field. These, in combination with heating of the samples, would also enable the separation of the volatile and non-volatile components. Coupled with characterization of the morphology and chemical composition of the emitted particles, either by online or offline instruments, this would provide useful information for fully characterizing the plumes and consequently assessing the impacts of the particles they contain on local air quality and human health. Finally, having additional instruments upwind of the runway could help better characterize background concentrations in cases where significant fluctuating particles sources are present upwind, providing also a way to better characterize plume dynamics in weaker cross-winds.

Overall, the measurements indicate high concentrations of very small particles affecting communities and dwellers in the vicinity of airports and airport employees. While these measurements were carried out ca. 200 m from the runway, we note that sample measurements performed at a distance of 1.5 km from the runway also indicated distinct plumes associated with individual aircraft operations, with peaks having values that are multiple times larger than the background number concentrations. Considering that such small particles can penetrate deep into the human respiratory system and cause adverse effects, such concentrations are likely to negatively affect human health. Further studies would be needed in order to assess human exposure to such high concentrations of sub-25 nm particles, and the associated risks involved. This is important as emissions from aircraft are likely to remain a concern for the coming decades, and increasingly so while other sources of emissions inside and in the vicinity of airports (e.g. Ground Support Equipment, surrounding road traffic) get electrified, especially as epidemiological studies increasingly highlight the importance of the number than the mass or volume concentrations of ultrafine particles. Recent studies^28,46–48^ indicate that the use of Sustainable Aviation Fuels will likely improve PM emissions from aircraft, but little is known on the implications of this for local air quality. Measurement efforts will continue to play a crucial role in better understanding these impacts. Long-term measurements at fixed locations, including the ongoing regulatory and other monitoring efforts in and around airports are and will continue to serve as the backbone in this respect. Despite the present short-comings (e.g., total PM, reduced accuracy), portable (cost-effective) sensors, like the ones utilized here, the measurements could complement the long-term air quality monitoring efforts, allowing for enhanced spatiotemporal resolution and covering different backgrounds and meteorological conditions.

## Methods

To capture the real-world plume from operational aircraft, we perform a series of field measurements downwind of an operational runway at Amsterdam Airport Schiphol using portable particle sensors. The measurements from the three particle sensors deployed are coupled to aircraft surveillance and meteorological data, and each aircraft operation is linked to an observed plume. We quantify the mass and number concentrations as well as the size of the particles in the resulting plumes, and analyze the differences among different operations, aircraft types, engine models, as well as the expected emissions performance using data provided by engine manufacturers in the ICAO Engine Emissions Data Bank^18^.

### Measurement setup and equipment

**Fig. 7:**
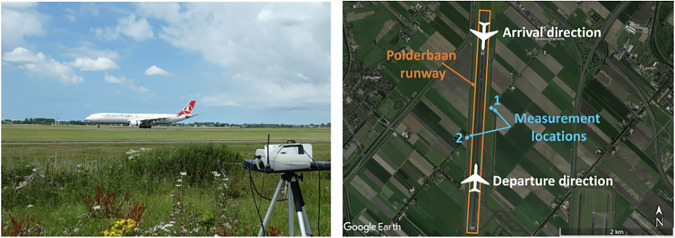
**Measurement setup.** Measurement setup downwind of the Polderbaan runway of Amsterdam Airport Schiphol.

Field measurements are carried out at Amsterdam Schiphol Airport (IATA: AMS; ICAO: EHAM), which has been ranked as the 11^th^ busiest airport globally in 2022. Amsterdam Schiphol Airport serves most international airlines globally and aircraft types representative of the global fleet^49^. This enables us to obtain observations from all common aircraft types and aircraft engine models in a consistent manner. The field measurements took place over five days, with the instruments operated 190 m downwind of the Polderbaan runway (18R/36L), which is one of the main runways of the airport, as shown in Fig. 7. We capture in total 603 operations (incl. 9 that involve ground vehicles), of which we are able to detect the plumes for 497, covering both aircraft departure and arrival operations, over different seasons. Table 1 summarizes the meteorological conditions in each of the five field measurement days.

**Table 1:** Overview of the operations and instruments employed during the different measurement days, and ambient meteorological condition ranges (07:00-17:00)

Date	Operation	Number of aircraft	Instruments present, and diluted as noted	Temperature (°C)	Relative humidity (%)	Wind speed (m/s)	Wind direction range (°)
15 July 2022	Arrivals	131	Partector 2, CPC, PM monitor	18–21	49.1–60.0	2.1–7.2	260–310
28 July 2022	Departures	160	Partector 2, CPC, PM monitor	16–21	48.8–63.1	4.6–6.7	40–90
22 December 2022	Arrivals	82	Partector 2 (diluted), CPC (diluted), PM monitor	5–10	87.4–100.0	2.1–6.7	140–270
15 March 2023	Arrivals	145	2 × Partector 2 (1 diluted), Partector 2 Pro, CPC (diluted), PM monitor	3–9	42.4–86.7	0.5–5.1	190–250
23 March 2023	Arrivals	76	2 × Partector 2 (1 diluted), Partector 2 Pro (diluted), PM monitor	11–15	62.7–81.7	7.7–12.3	220–240

Three types of portable particle sensors are deployed to characterize the particle concentration and size of the aircraft plumes: a multi-metric nanoparticle detector (Naneos Particle Solutions GmbH, Switzerland, mods. Partector 2 and Partector 2 Pro), a portable condensation particle counter (CPC, TSI Inc., USA, mod. 3007^37,38^), and a custom-made PM monitor that employs a low-cost Optical Particle Counter (OPC)^39^. A summary of the instruments deployed for each day of the field measurements is provided in Table 1. A brief description of these instruments is also provided below, while more details can be found in the SI.

Partector 2 is a small, light (415 g), battery-operated multi-metric monitor, which reports the number concentration, and the average diameter of particles in the size range of 10–300 nm (ref. ^50^) and the lung deposited surface area (LDSA) (see SI Section S1.1 for more details). It measures number concentrations up to 10^6^ particles/cm^3^. In some measurements, we also used Partector 2 Pro, which additionally provides an 8-channel size distribution in the 10–300 nm range. Both instruments were operated with an inlet flow rate of 0.5 l/min and a sampling frequency of 1 Hz. The counting and sizing (average particle diameter) accuracy of the instrument is ±30% (whichever is higher) when sampling aerosols with number concentrations up to 10^6^ particles/cm^3^, as reported by the manufacturer^50^ and in the literature^51,52^. Since the individual plumes observed in some cases have number concentrations that approach or exceed the 10^6^ particles/cm^3^ threshold (set by the manufacturer), a simple diluter is also used in some of the measurements.

The CPC used in our study provides the number concentration of the sampled aerosol particles, having sizes in the range from ca. 10 to 1000 nm, by first growing them via condensation of isopropyl alcohol to micron-sized droplets, and subsequently detecting/counting them by optical methods. This CPC has a nominal counting accuracy of ±20% up to 10^5^ particles/cm^3^ according to the manufacturer^53^ (see SI Section S1.2 for more details). The measured plume number concentration peaks from aircraft regularly exceed the upper instrument concentration threshold, so a diluter was also used in some of the measurements. For the measurements where the diluter was not present, the CPC measurements are only used to determine the plume width and not the peak values. The CPC was compared with another bench-type CPC, which was used as a reference to verify its proper operation prior to the measuring campaign. In addition, its operation was regularly checked during the campaign by performing zero concentration tests (i.e., sampling from a HEPA filter), measuring the flow rate and comparing the reported number concentration with the Partector 2, when sampling indoor aerosol.

The PM monitor used in our measurements employs a low-cost Optical Particle Counter (OPC) in a similar way to that described by Bezantakos et al.^39^ (see SI Section S1.3 for more details). The PM monitor reports the number size distribution of particles in the size range between 0.30–12.4 μm at 16 different size bins, with a sampling rate of 20 samples/min. In addition, the cost-effective PM monitor reports PM_1_, PM_2.5_, and PM_10_ mass concentrations, assuming an apparent particle density of 1.6 g/cm^3^.

### Aircraft and engine data

Aircraft activity data is obtained from the OpenSky network, which provides crowdsourced Automatic Dependent Surveillance–Broadcast (ADS-B) data at ca. 1 s resolution^28,46–48,54^. By grouping the ADS-B data by ICAO24 code, a unique code coupled to each vehicle transmitting ADS-B, and separating it by the flight-specific callsign, we classify tracks as arrivals, departures, and taxiing operations. This allows us to construct a timeline of runway-specific aircraft operations. The timing in the aircraft activity data is crucial for linking individual aircraft to recorded plumes (as explained in subsection ‘Data processing’). In case the aircraft activity is incomplete and for validation purposes, continuous timelapse photographs during the measurements are additionally recorded using a GoPro Hero 3 (Black Edition) camera. These pictures are, in part, used to support the ADS-B aircraft activity data.

The specific engine model the aircraft is equipped with has been shown to have significant consequences for the resulting emissions^10^. For almost 80% of the flights, aircraft-engine type matching was obtained from OpenSky, which retrieves this data from national aircraft registries, among other sources^54^. For the remaining ca. 20% of the flights, OpenSky did not specify an engine type, in which cases an engine type was manually added for a flight based primarily upon data from the Dutch Aircraft Registry and a remaining small number from an online Aircraft Registration Database Lookup^55,56^. The ICAO Aircraft Engine Emissions Databank (version v29B) specifies the emission indices (corrected for system losses) for common aircraft engine models and engine thrust settings, which we use to compare against the emissions performance we measure^57^.

### Meteorological data

Meteorological conditions, which can affect plume dynamics, dispersion, and the resulting concentrations, are obtained from the Meteorological Terminal Aviation Routine Weather Reports (METAR), retrieved from the Iowa State University environmental mesonet^58^. These contain the temperature, pressure, humidity, wind speed, and wind direction at Amsterdam Airport Schiphol (normally) every 30 min. The weather station providing these measurements is located ca. 6 km from the Polderbaan runway^59^.

### Data processing

The measured particle timeseries, the aircraft surveillance data, and the meteorological data are jointly analyzed to assign the recorded plumes to individual aircraft operations and characterize their properties. The measurements are quantitatively analyzed for the Partector 2 and the CPC, and qualitatively discussed for the PM monitor, as individual aircraft plumes were not always evident in the latter (see SI Section S1.2 for more details).

The Partector 2 and the CPC measurements are processed as follows. When a diluter is used, the recorded particle concentrations are first corrected for the dilution ratio (using the measured flow rate through the diluter). The signal from the instrument is split into two parts: a background signal and a local pollution signal. The background signal is obtained by calculating the 5^th^ percentile on a rolling window basis of 5 minutes. The local pollution signal is the difference between the background signal and the total signal. We note that since the total signal is orders of magnitude larger than the background signal, the background correction does not meaningfully affect the aircraft-specific contribution results. The local pollution signals for the Partector 2 and CPC are smoothed using a moving average filter of 15 samples (i.e., 15 seconds), and 12 samples for the Partector 2 Pro due to its lower sampling rate. The aircraft activity time series and the cross-wind component of the wind velocity allow the estimation of the anticipated plume travel from the runway to the measurement location. A plume (in the “local pollution signal”) is coupled to an aircraft movement if the actual plume start time is shifted by less than 30 s from the estimated plume arrival time of each aircraft. Using both the ADS-B data as well as the runway timelapse photographs, we disregard the plumes from operations that coincide with the presence of other combustion sources in the immediate vicinity (e.g., airport patrol vehicles passing by, etc.). We note one outlier plume that was erroneously coupled to a flight, which we removed manually from the final data set. For each recorded plume, we calculate the plume peak (in #⋅cm^−3^), the plume width (in s), and the area under the recorded plume curve, referred to as plume area (in #⋅cm^−3^⋅s). We consider the latter to be a more robust metric for comparing plumes measured under different conditions (e.g., wind speed and direction), which we also verify with simulations (see Section ‘Plume simulations’, and Section S2 in the SI).

**Fig. 8:**
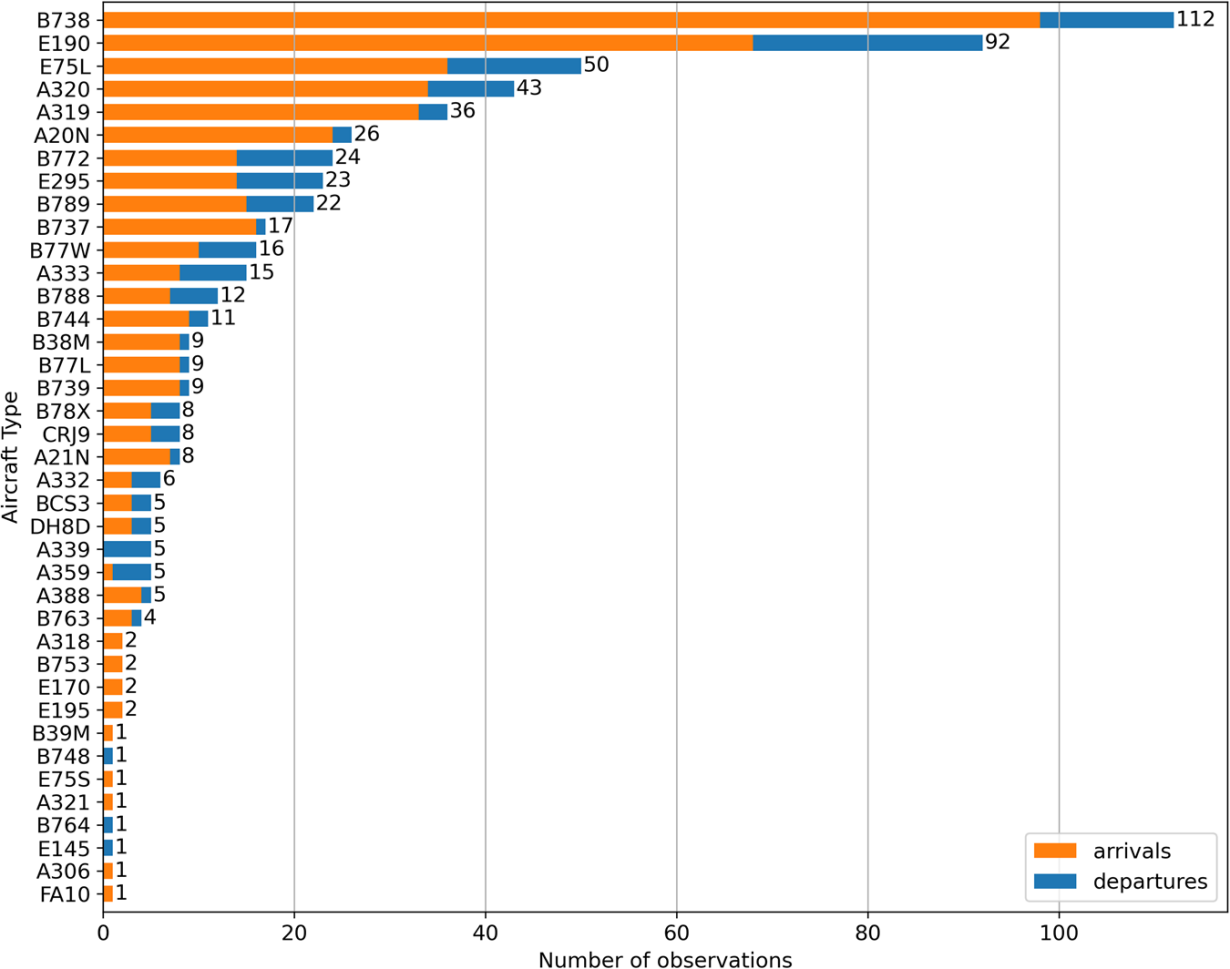
**Overview of observed aircraft types**. Aircraft types observed, per operation, over all measurement days.

While we capture aircraft types from the entire global fleet, since AMS is the hub of the Dutch Royal Aviation Company KLM, we have an increased representation of aircraft present in its current fleet (including its subsidiaries, KLM Cityhopper and Transavia). As shown in Fig. 8, these are the aircraft models B738, B772, and B789 manufactured by Boeing, USA, and E190 manufactured by Embraer, Brazil.

### Plume simulations

We complement the measurements with a set of plume simulations to a) compare the physical dimensions of the plume with the measured ones, and b) evaluate the suitability of different plume metrics (e.g. plume area) for the comparison between plumes probed under different conditions (e.g. wind speed and direction). 2D simulations are performed with COMSOL Multiphysics version 6.2 using the High Mach Number Flow interface coupled with the Transport of Diluted Species module. COMSOL uses the Discontinuous Galerkin (DG) methods with nodal discontinuous Lagrange shape functions as the basis functions, and an explicit Runge-Kutta method for the time integration^60^. We use the Spalart-Allmaras turbulence model and dilute an initial passive tracer concentration (in this case, CO_2_) of 200 ppm to a background with no concentration, in order to track the physical plume properties.

The plume simulations focus on arrivals and departures of B738 aircraft, for which we have the most measurements. The 2D setup includes two jets of that are 1.55 m in diameter (corresponding to that of the CMF-56 engine^61,62^), placed at a distance of 5.175 m from each other (corresponding to the B738 architecture). The engines are placed and the edge of the computational domain, which corresponds to an area surrounding the runway of dimensions 1 km perpendicular to the runway by 10-40 km along the runway, depending on the crosswind speed. The velocity reference frame is fixed on the aircraft, and thus, the aircraft speed is included in the axial component of the wind speed. We calculate the plume characteristics (peak in mol/m^3^, width in m, area in mol/m^2^) at a distance of 190 m laterally from the runway, which is the distance between the runway and the point of our measurements, and compare the plume width with equivalent values from the measurements (width in s), correcting with an average cross-wind value to harmonize the modeling units. More details about the simulations are provided in Section S3 of the SI.

## Supplementary information

Supplementary Information

## Data Availability

The measurement data obtained and presented in this work is made publicly available at 10.4121/38fa4b11-2854-4e76-ae57-e7463a049933.
