# Supplementary material for: Aircraft emissions of ultrafine particles characterized by real-world near runway measurements
Source: NPJ Clim Atmos Sci. 2025 Jun 19;8(1):232. doi: 10.1038/s41612-025-01095-9 (PMC12176625; doi:10.1038/s41612-025-01095-9)
Supplement: Supplementary file 1 — Supplementary Information [file 41612_2025_1095_MOESM1_ESM.pdf]

Supplementary Information: Aircraft particle emissions characterization determined by real-world near runway measurements

Jeff Maes[1], Spyros Bezantakos[2], Luccas K. Kavabata[1], George Biskos[2,3], Irene C. Dedoussi[1,4]

[1] Aircraft Noise and Climate Effects section, Faculty of Aerospace Engineering, Delft University of Technology, Delft 2629 HS, the Netherlands

[2] Climate and Atmosphere Research Center, The Cyprus Institute, Nicosia 2121, Cyprus

[3] Faculty of Civil Engineering and Geosciences, Delft University of Technology, Delft 2628 CN, the Netherlands

[4] Department of Engineering, University of Cambridge, Cambridge CB3 0DY, United Kingdom

Corresponding author: Irene C. Dedoussi (icd23@cam.ac.uk)

Contents:

S1 Additional instrument details 2

S1.1 Partector 2 and Partector 2 Pro 2

S1.2 CPC 2

S1.3 Custom OPC 3

S1.4 Ability to detect individual aircraft plumes 4

S2 Variability of plume metrics under different meteorological conditions 6

S3 Plume simulation specifics 7

S4 Effects of instrument performance and uncertainty 12

S5 Particle size distribution for individual aircraft types 17

S1 Additional instrument details

S1.1 Partector 2 and Partector 2 Pro

The Partector 2 and Partector 2 Pro (Naneos Particle Solutions GmbH, Windisch, Switzerland) are portable (14 × 8.8 × 3.4 cm; 415 g) monitors which report LDSA, average particle diameter and number concentration of aerosols in the size range from 10 to 300 nm. In addition, the Partector 2 Pro classifies the sampled aerosols in 8 size bins, making it possible to derive their size distribution. Both are battery equipped with autonomy reaching around 20 hours. The instruments' operating principle is based on the measurement of the current induced by charged particles of the same polarity, which is proportional to their LDSA[1]. In addition the induced current is proportional to the product of the number concentration and average charge per particle, which in turn depends on its size[2,3]. Both instruments employ pulsating (i.e., on-off) unipolar corona chargers and two open path electrometers in series separated by an electrostatic precipitator, which acts as a low pass electrical mobility filter. The first electrometer measures the current induced by all sampled and charged particles, while the subsequent one (i.e., downstream of the precipitator) measures the induced current of particles that passed through the precipitator (i.e., those with reduced electrical mobilities). In Partector 2, the electrostatic precipitator operates at a constant voltage, thus removing particles with electrical mobilities higher than a specific threshold. By comparing the signals of these two electrometers and assuming a polydisperse size distribution with a geometric standard deviation of ca. 1.9, one can derive the number concentration and average size of the sampled aerosols. In the Partector 2 Pro the electrostatic precipitator switches between 4 different voltages, thus removing particles with electrical mobilities higher than 4 thresholds. In this case, comparing the signals obtained from its two electrometers at all precipitation voltages one can derive the number of particles at 8 different size bins[4]. Both instruments have an inlet flow rate of 500 cm^3/min, automatically regulated by an internal pump. Their accuracy remains unaffected by the ambient temperature conditions encountered in this study, but can deteriorate under specific conditions related to the particle number concentration and size distribution width (see section S4 herein for more details).

S1.2 CPC

The Model 3007 CPC by TSI (TSI Incorporated, Shoreview, MN, USA) is a battery-powered hand-held (29.2 × 14 × 14 cm; 1.7 kg) device. It uses isopropyl alcohol as the operating liquid and has an inlet flow rate of 700 cm^3/min, from which 100cm^3/min pass through the saturator, condenser and optical detector. The instrument's standalone endurance is around 6 hours of continuous operation, defined by the battery pack capacity and the wick's isopropanol quantity. Longer operation periods require refilling of the wick with isopropanol and battery replacement (around 15 minutes of turnover time). The CPC exhibits a 50% detection efficiency (D_{50}) at ca. 10 nm, which increases to 100% above ca. 20 nm in laboratory conditions[5]. The instrument's detection efficiency curve is affected by ambient temperature conditions, exhibiting enhanced detection efficiencies of aerosols smaller than 10 nm (i.e., reduction of D_{50}) at temperatures lower than 20 °C and vice versa at temperatures above this threshold[6]. Specifically, for temperatures in the range of 15 to 21 °C the detection efficiency curve of the CPC remains almost unaffected. For temperatures close to 10 °C, the CPC's D_{50} reduces to ca. 8 nm (i.e., increased detection of particles <10 nm), while it exhibits a 100% detection efficiency for all aerosols above 18 nm. Close to 5 °C the CPC's D_{50} reduces to 6.5 nm,

while it exhibits a 100% detection efficiency for particle sizes above ca. 14 nm. Given the ambient temperature ranges in which the measurements were conducted (see Table 1 in the manuscript), the CPC's detection efficiency curves were close to the nominal ones during July and slightly affected during the 23rd of March. During the 15th of March and the 22nd of December (i.e., prevailing temperatures below 10 °C) the CPC would exhibit a slight enhancement of its detection efficiency for particles smaller than 20 nm. The maximum detectable size is limited at ca. 1000 nm, due to the geometry of the instrument (i.e., bigger particles impact on bends of the instrument's flow path).

S1.3 Custom OPC

The custom-made, cost-effective optical particle counter (OPC) was built around the low-cost Alphasense R1 sensor (Ametek Sensor Technology House, Essex UK), which measures the size distribution of aerosols bigger than 300 nm. The low cost sensor measures the scattered light resulting from illuminating sampled particles with a monochromatic source. The amplitude of the scattered light is proportional to the size of the illuminated particle but depends on its optical properties (i.e., refractive index), which in turn depend on its chemical composition. In such instruments (i.e., OPCs) a refractive index is assumed for inverting the raw measurements (i.e., light amplitude) to particle size. In the case of the Alphasense R1 sensor a refractive index of 1.5 is assumed, while the sensor comes pre-calibrated with a help of a reference optical instrument (TSI 3330 OPS; TSI Inc., USA). The internal firmware performs the inversion of raw measurements providing results of counts per size bin over 16 different size channels covering a range from 0.30 to 12.4 μm. In addition, the firmware calculates the mass concentration of the sample particles in three fractions; namely PM_1, $PM_{2.5}$ and PM_{10} which correspond to the mass concentration of particles smaller than 1, 2.5 and 10 μm respectively. For converting the number size distribution to mass concentrations, the firmware assumes an apparent density of the sampled aerosols equal to 1.6 g/cm^3. The low cost sensor is equipped with a small fan for achieving a typical flow rate of around 240 cm^3/min through its detection volume. However this fan does not achieve the necessary pressure for overcoming pressure drops induced by upstream piping and/or aerosol pretreatment devices (e.g., dryers, cyclones etc.). In addition, the flow rate of the fan equipped sensor and consequently its performance may be affected by wind bursts. For these reasons Bezantakos et al. (2021)[7] modified and tested another low-cost, fan-equipped optical sensor from the same company (i.e., Alphasense model OPC-N2). The modification included the replacement of the sensor's fan with a pump and a critical orifice for maintaining a constant flow rate under all sampling conditions. The modified sensor exhibited similar performance to that of the unmodified one during laboratory tests with polystyrene spheres (i.e., monodisperse particles of known refractive index, commonly used for testing and calibrating optical particle sizers). Most importantly, the performance of the modified sensor was comparable to that of a laboratory grade OPC (TSI 3330 OPS; TSI Inc., USA) when both instruments were sampling the same polystyrene spheres having total number concentrations below 1000 #/cm^3. As a follow up from this study, we built a cost-effective instrument which combines the advantages of modifying the flow system of a low cost optical sensor with all the necessary auxiliaries (i.e., power, data acquisition and storage, screen etc.) systems necessary for its operation, data visualization and storage. The cost-effective instrument saves the measurements as counts per size bin, which can be converted to number concentration by accounting for the sample flow rate through the detection volume (i.e., 336 cm^3/min) and illumination period (0.63 seconds). It also saves the mass concentrations of PM_1 , $PM_{2.5}$ and PM_{10} which are calculated by the sensor's firmware as described above.

S1.4 Ability to detect individual aircraft plumes

As presented in the main manuscript, with the Partector 2 and the CPC we are able to clearly detect plumes following the aircraft activity. Out of the total ~600 operations (including primarily fixed wing aircraft but also other vehicles) recorded in the ADS-B activity data during the measurement days, we are able to clearly characterize 75% of the plumes with the Partector 2. Due to the CPCs maximum number concentration threshold, which was regularly exceeded when the flow was undiluted, we utilized a smaller number of CPC measurements for characterizing aircraft plumes. However, the number of useful CPC-data significantly increased when the diluter was used, since in this case the CPC-reported number concentrations were similar to those of the Partector 2.

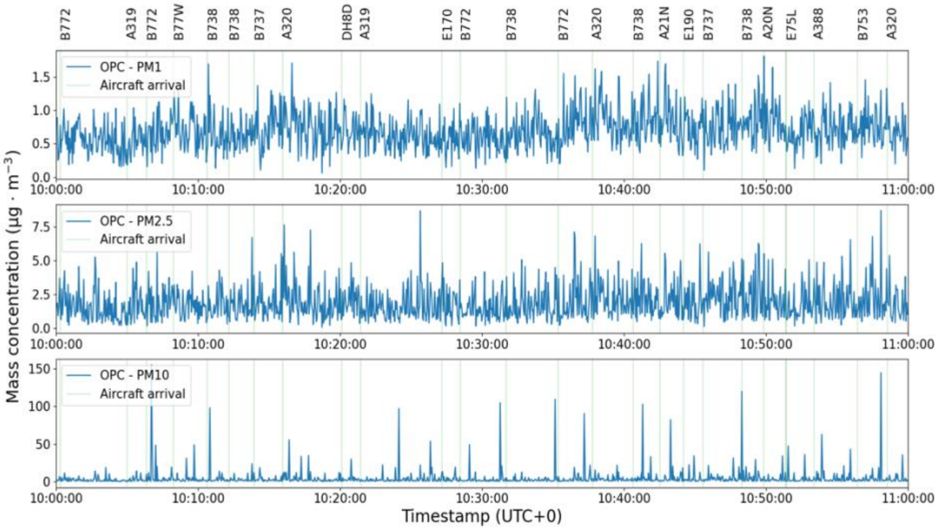

(a) arrivals

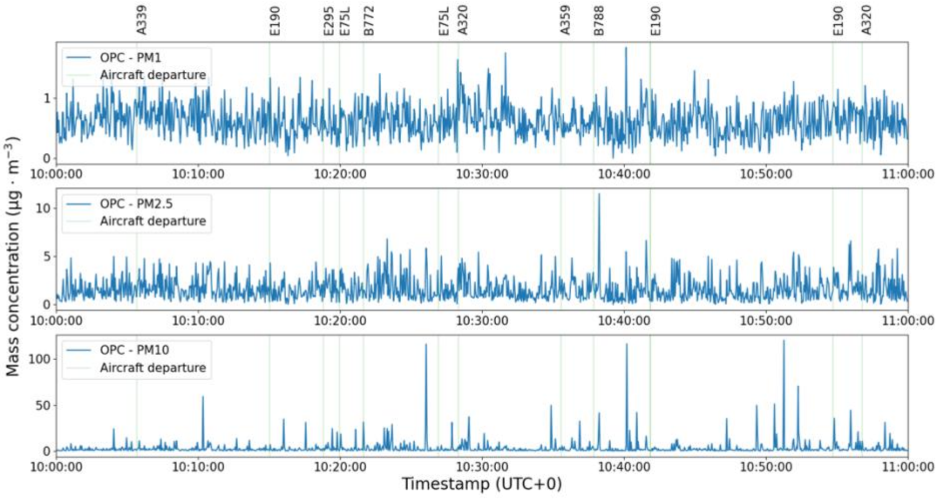

(b) departures

Figure S1: Sample timeseries of measured signals for 1-hour during an arrival (a) and a departure (b) measurement day as recorded by the custom OPC. The plot rows from top to bottom display PM_1 (top),

PM$_{2.5}$ (middle) and PM$_{10}$ (bottom) mass concentration (in $\mu g \cdot m^{-3}$) derived using the PM monitor. The instrument is undiluted. The light green vertical lines show the aircraft type departing or arriving.

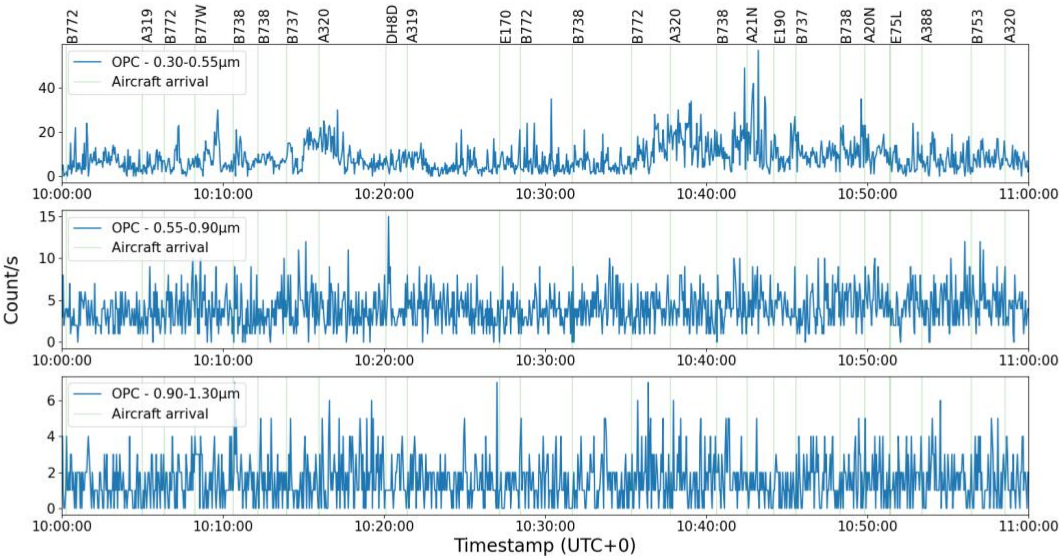

(a) arrivals

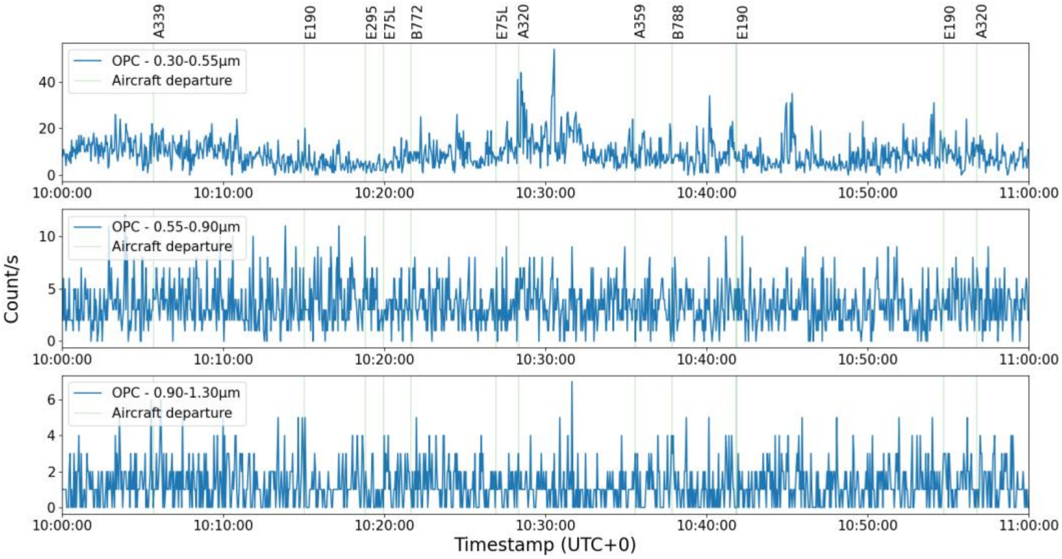

(b) departures

Figure S2: Sample timeseries of measured signals for 1-hour during an arrival (a) and a departure (b) measurement day as recorded by the PM monitor. The plot rows from top to bottom display the custom OPC 0.30-0.55 µm bin (top), the 0.55-0.90 µm bin (middle) and the 0.90-1.30 µm (bottom) particle count rate (in particles·m-3·s-1). The instrument is undiluted. The light green vertical lines show the aircraft type departing or arriving.

As discussed in the main manuscript, the timeseries of the PM monitor did not reveal individual aircraft plumes as clearly as those identified by the Partector 2 and the CPC. Corresponding timeseries samples for the PM monitor are shown in Figure S1 and Figure S2. While the largest variations are present in the $PM_{2.5}$ and the smallest size bin (300-550 nm) signals, these are not consistently aligned with individual aircraft operations, while in many cases they cannot be distinguished from the background signal. While the PM monitor was present in all measurement days, we do not include it in the analysis presented in the main manuscript since it was not able to provide information on an individual aircraft basis.

As a result, we focus our analysis and discussion on the results obtained by the Partector 2 and Partector 2 Pro instruments, while using the data from the diluted-flow CPC to provide a comparison for the Partector 2 measurements. While the measurements that are presented in this work took place at a distance of ~200 m laterally from the runway, we note that the Partector 2 and the CPC were also able to identify individual plumes also at a distance of 1.5 km laterally away from the runway. In those cases, and as expected, the plumes were wider with lower peaks in number concentrations, and as a result a diluter was not necessary for the CPC.

S2 Variability of plume metrics under different meteorological conditions

The most commonly observed aircraft in our measurements is the B738. For this aircraft Figure S3 presents its different plume metrics under different cross-wind conditions and Figure S4 presents its measured plume particle concentration area under different meteorological conditions.

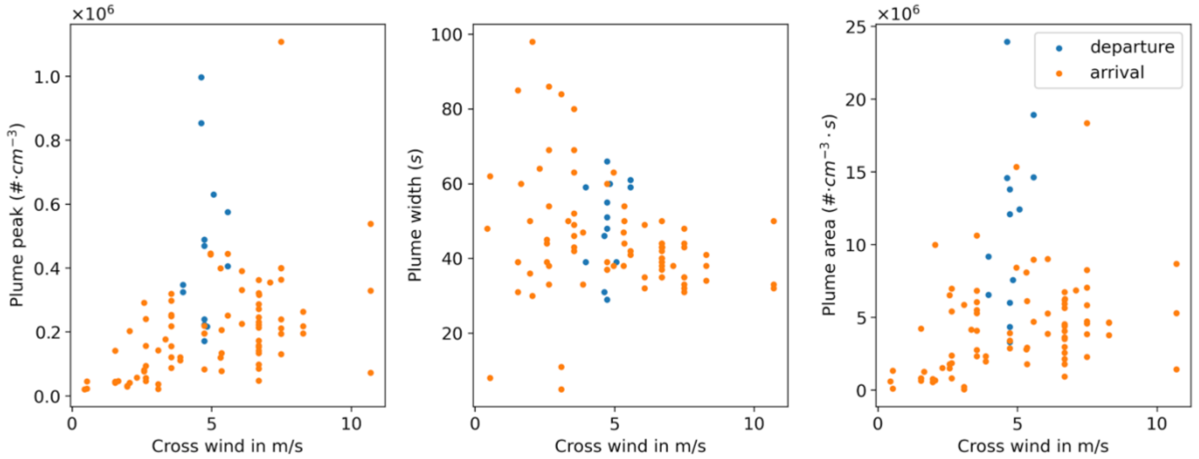

Figure S3: Partector 2 measured plume peak (left), width (middle), and area (right) for the B738. Includes 96 measurements.

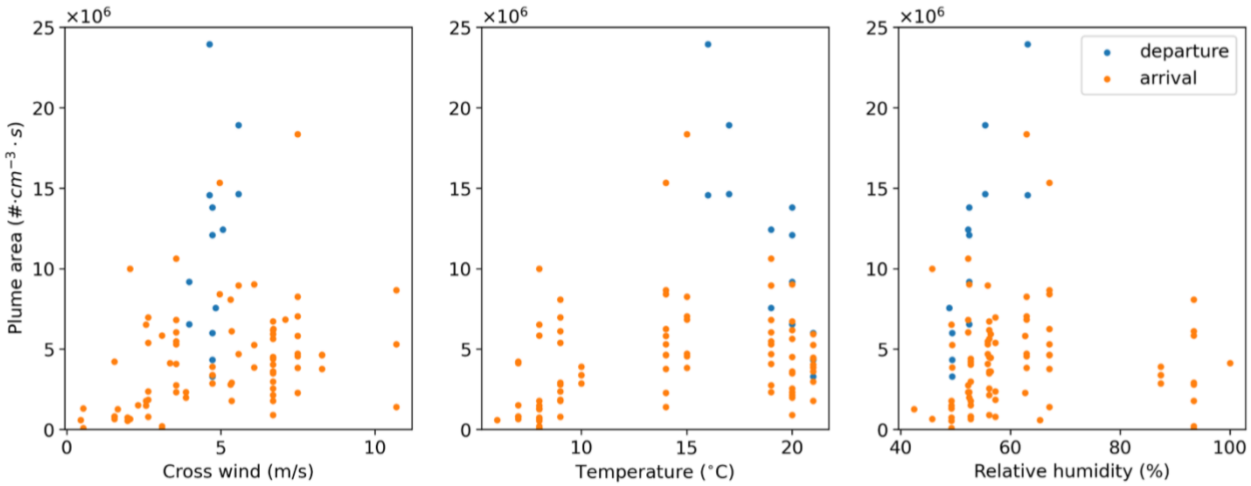

Figure S4: Partector 2 measured plume area (in #·cm^{-3}·s) under different ambient temperature (left) relative humidity (middle) and wind conditions (right) for 112 observations of the B738 (departures in blue, arrivals in orange).

S3 Plume simulation specifics

As presented in Section 'Plume simulations' in the main manuscript, COMSOL simulations were performed to support the measurements and their analysis. Further details on the simulation setup and the analyzed output are provided here.

Two jet simulations based on averaged values of the aircraft velocity, wind speed, wind angle, and ambient temperature measured for the Boeing B738 from data for departures and for arrivals were performed. These are two dimensional compressible simulations with the Sparlat-Allmaras turbulence model and the transport of diluted species to simulate dispersion of CO_2 in the plume, in a background atmosphere (without CO_2). We use as a proxy to estimate plume dimensions. The computational domain corresponds to 40 by 1 km for crosswind 1 m/s, 30 by 1 for crosswind 2 and 3 m/s, and 10 by 1 for crosswind from 4 to 10 m/s along and perpendicular to the runway, respectively. Figure S5 illustrates the computational domain and the concentration field for the B738 in departure configuration. It can also be seen that the plume aligns with the wind angles also expected from literature[8]. Since the computational domain under consideration is quite large, a mesh refinement technique was used to refine the grid in the plume direction.

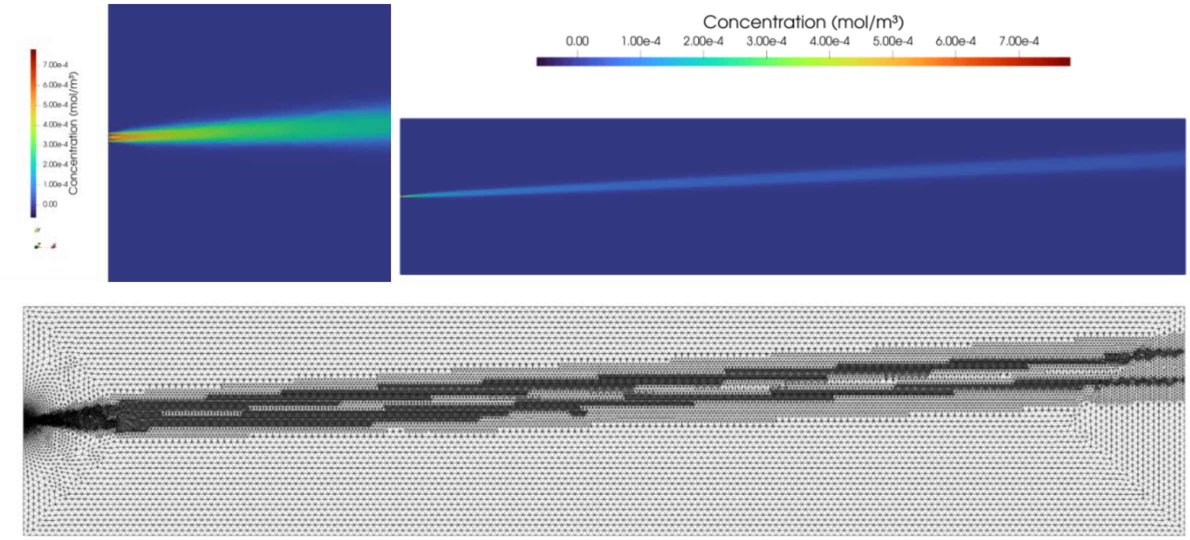

Figure S5: Detailed view of the two-jet configuration (top left) and an overview of the concentration field in mol/m^3 (top right), and computational grid after adaptive mesh refinement (bottom), for the B738 for a departure configuration of the entire computational domain for a crosswind of 4 m/s.

The jets are placed at a distance of 5.175 m from each other, which corresponds to the distance between the B738 engines. The reference frame is fixed on the aircraft and the aircraft speed is included in the axial component of the wind speed, similarly to the approach of Barrett et al. (2013)[8], according to

$$U_{wind} = (U + U_{plane})e_x + Ve_y$$

where U denotes the axial component of the wind velocity, U_{plane} denotes the aircraft velocity, and V denotes the crosswind, in the axial (e_x) and cross-wind (e_y) direction.

The simulation parameters are summarized in Table S1 for both the departure and arrival simulations. An exhaust pressure of 161.4 kPa was used in both cases.

Table S1: Simulation parameters for departures and arrivals.

	Aircraft velocity (m/s)	Wind speed (m/s)	Wind angle (degrees)	Ambient temperature (K)	Exhaust temperature (K)	Initial concentration (ppm)
Departures	81.79	5.73	62.14	292.22	841.53	200
Arrivals	62.75	6.13	240.60	286.80	841.53	200

The plume measurements were performed at a distance of 190 m laterally from the Polderbaan runway of Amsterdam Airport Schiphol. To be able to compare with the measured plume parameters (e.g. width), we are interested in the concentration of the simulated plume at the same distance in the horizontal line y = 190 meters (schematic in Figure S6). We note that the plume width varies depending on the plane in which it is

measured. To investigate this, the plume properties were taken with respect to the line perpendicular to the middle point (x_m) between points x_1 and x_2, which are the initial and final points where the plume crosses the line y=190 mm (at a magnitude of 5% of the plume peak to remove numerical noise), respectively, as also shown in Figure S6. For example, Figure S7 shows the concentration in mol/m^3 along the length of the plane perpendicular to the plume direction and passing through point x_1.

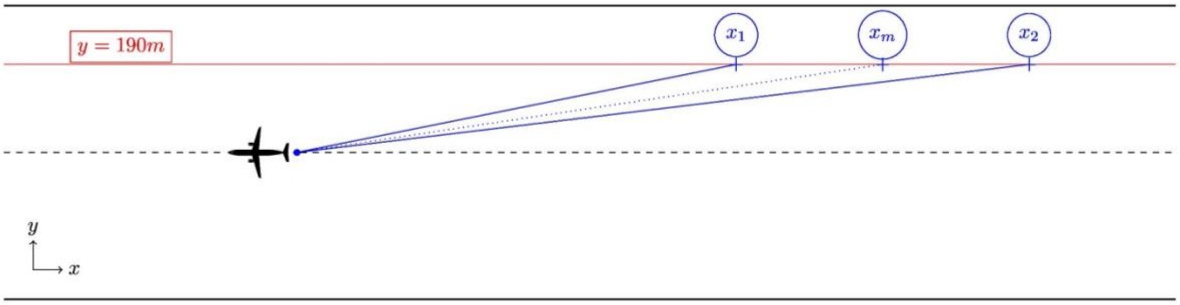

Figure S6: Schematic representation of the aircraft exhaust plume. The red line indicates the line y=190 m, the blue line represents the exhaust plume, and the points x_1, x_2, and x_m represent the initial, final, and middle points where the plume crosses the horizontal line y=190m, respectively.

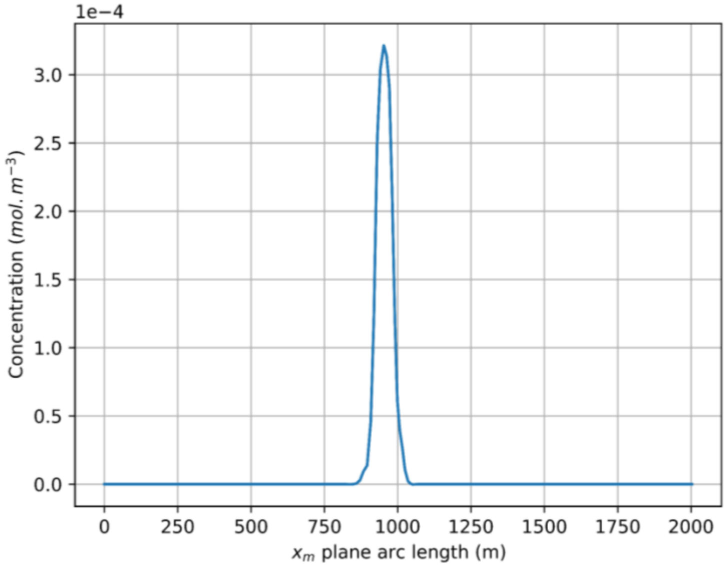

Figure S7: CO_2 concentration plot in the plane perpendicular to the plume direction and passing through point x_m.

In the main manuscript we present results from the x_m point, and here we discuss how this varies depending on this choice. We note that the area remains largely constant (within 6%) between the three points, whereas the peak decreases as the width increases further down the simulated plume.

Table S2: Simulated plume properties (peak, width, area) of three planes perpendicular to the plume direction. For comparison, as presented in the manuscript, the average departure plume width was 230 m (standard deviation 59 m) and the average arrival plume width was 183 m (standard deviation 74).

	Location	Peak ($\times 10^{-4}$ mol/m^3)	Width (m)	Area ($\times 10^{-2}$ mol/m^2)
Departure	$n(x_1)$	2.47	187.99	2.21
	$n(x_2)$	1.21	386.53	2.25
	$n(x_m)$	1.56	307.20	2.25
Arrival	$n(x_1)$	3.31	173.64	2.70
	$n(x_2)$	1.91	293.99	2.79
	$n(x_m)$	2.36	246.72	2.76

A set of 10 simulations were performed to evaluate the sensitivity of the plume properties with respect to the crosswind, as presented in Figure 8 of the main manuscript for x_m. Figure S8 presents the corresponding figure for all three locations.

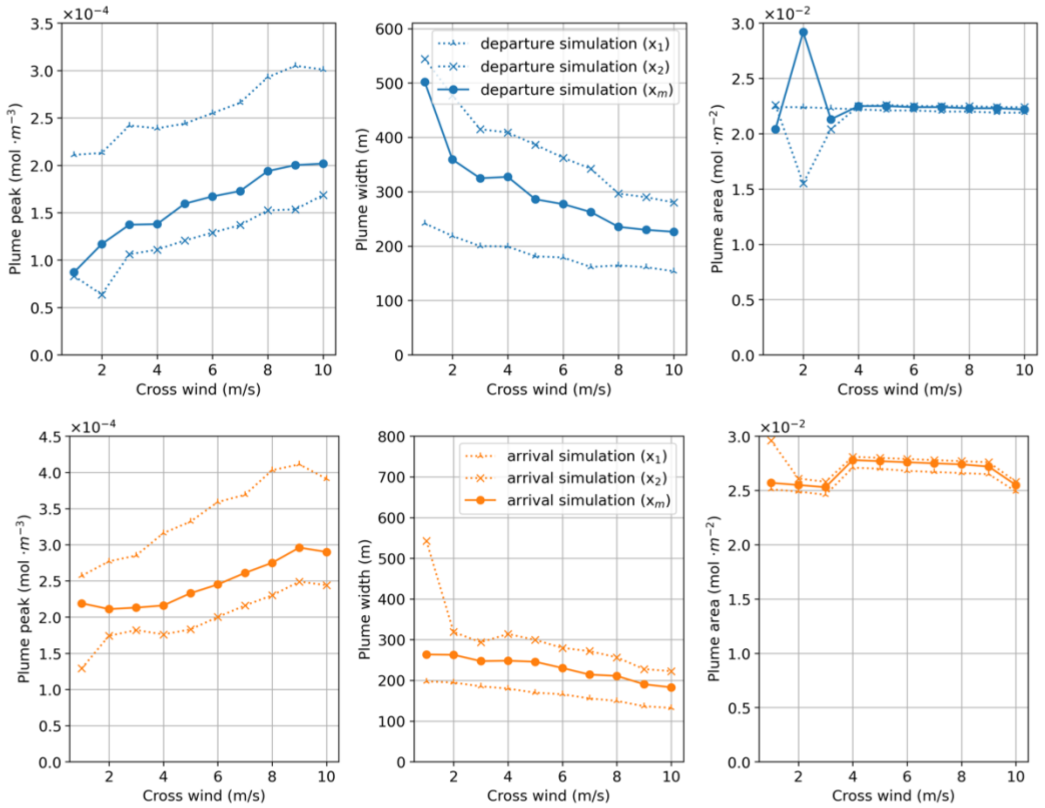

Figure S8: Plume height (mol/m³), plume width (m), and plume area (mol/m²) versus crosswind plots for the line normal to the plume direction at point x_1, at point x_2, at the midpoint between x_1 and x_2 (solid curve), for departures (top) and arrivals (bottom).

S4 Effects of instrument performance and uncertainty

The main manuscript presents both diluted and undiluted Partector 2 measurements jointly. Here we present the same plots separated in diluted and undiluted sampling flow conditions, including a discussion on how the dilution may affect the Partector 2 instrument performance. Only arrivals are presented as there are no diluted departure measurements available, but we note that the dilution might affect the departure measurements more substantially given the higher peak concentrations. Overall, while Figure 2 and Figure 3 in the main manuscript depict the calculated plume area using both diluted and undiluted concentration measurements, we note that the overall conclusions are not largely impacted by this choice, despite that peak concentrations of individual measurements can differ depending on whether the diluter was being used.

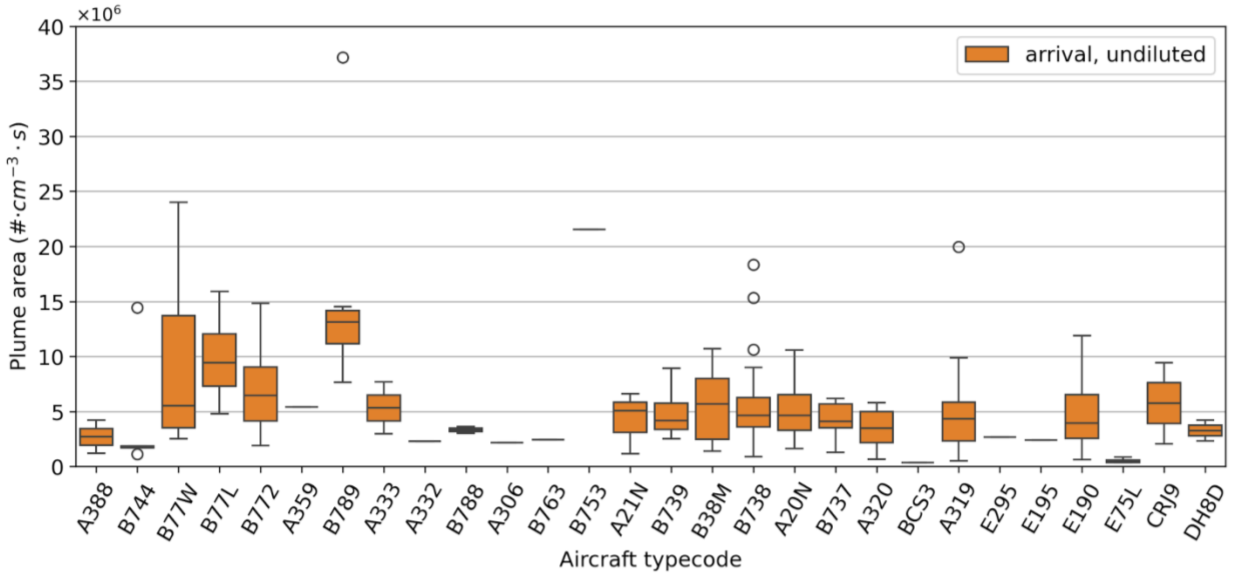

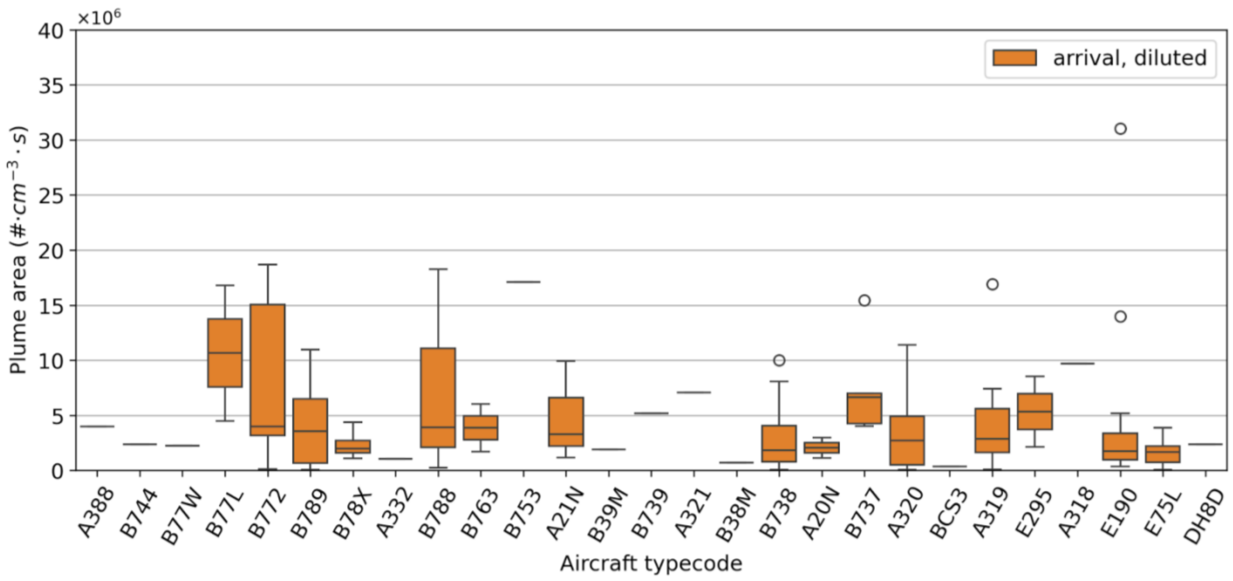

Figure S9: Particle number emissions performance for different aircraft types sorted by decreasing Maximum Take-Off Weight (MTWO), from larger (left) to smaller (right) aircraft during arrivals. Partector 2 measured plume area (in #·cm^{-3}·s) is shown without dilution and with dilution and corrected for the dilution ratio.

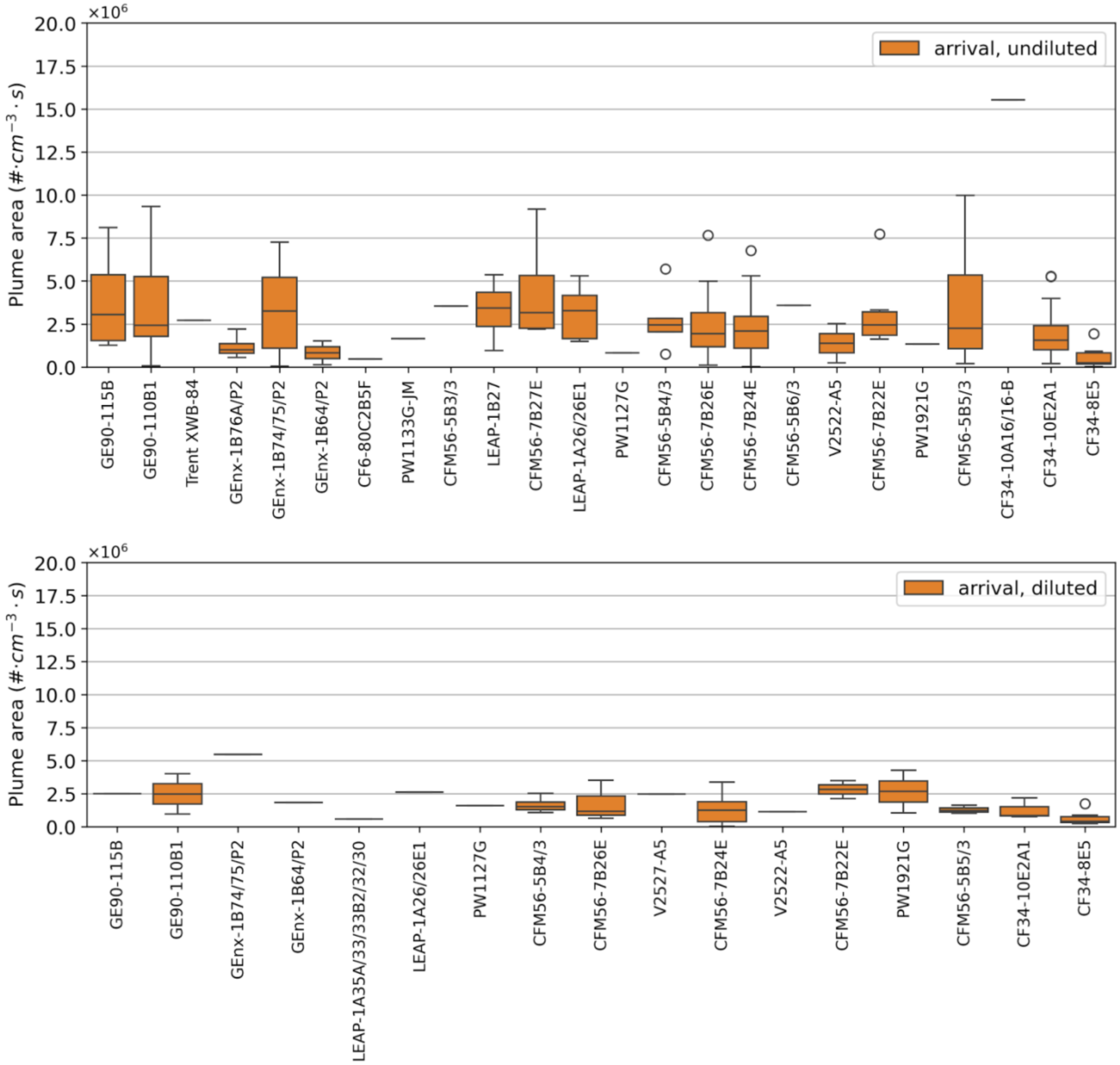

Figure S10: Particle number emissions performance for different engine models sorted by decreasing maximum rated thrust for arrival operations. The figures display the Partector 2 measured plume area (in $\#\cdot cm^{-3}\cdot s$) for aircraft powered by each engine scaled by the number of engines for undiluted measurements, and diluted and corrected for the dilution ratio.

Some of the presented variability is expected to originate from the instruments deployed, given their accuracies (i.e., 20% for the CPC and 30% for the Partector 2s; reported by the manufacturers). However, we should note that the above mentioned accuracies hold within the concentration operational envelope of the instruments (i.e., up to 10^5 and 10^6 $\#/cm^3$ for the CPC and the Partector 2s, respectively). Exceeding the maximum concentration threshold can reduce the accuracy of each instrument, resulting for instance in underestimating the actual particle number concentration in the case of CPC. Underestimating the particle number concentration would consequently result in underestimating the calculated plume area. Coincidence

counting (i.e., more than one particles illuminated and/or detected simultaneously or in shorter period than the processing time of the counting electronics) will start deteriorating the performance of the CPC at number concentrations above its maximum concentration threshold[5], as also shown in Figure 6 in the main manuscript. The accuracy of the Partector 2 can also deteriorate at very low or very high concentrations, depending on the signal of its two electrometers (see SI Section S1). At very low concentrations, the electrometer signals are strongly affected by the baseline noise of the electronics, while at very high concentrations (i.e., exceeding the instrument's maximum concentration threshold) the flux of aerosols will be comparable to that of the ions produced by their unipolar corona chargers (i.e., ion depletion). This has the effect of reducing the average number of charges per particle, in comparison to those that the instrument has calibrated with in order to derive the number concentration and average size. In addition, Partector 2 derives the average particle diameter and number concentration, assuming that the sampled particles exhibit a lognormal distribution with a geometric standard deviation of 1.9. Therefore, reduced accuracy is expected in the case that the size distribution of the sampled particles differs significantly from that assumed during the calibration of the instrument.

Finally, while we correct for background PM concentrations and presence of non-aircraft sources by subtracting the time-averaged number concentration as detailed in subsection 'Data processing' in the main manuscript, we do not account for their different size distribution. However, we note that these are not likely to strongly influence the aircraft-attributable number concentration as the ambient concentrations measured are on average multiple orders of magnitude lower than the observed plumes.

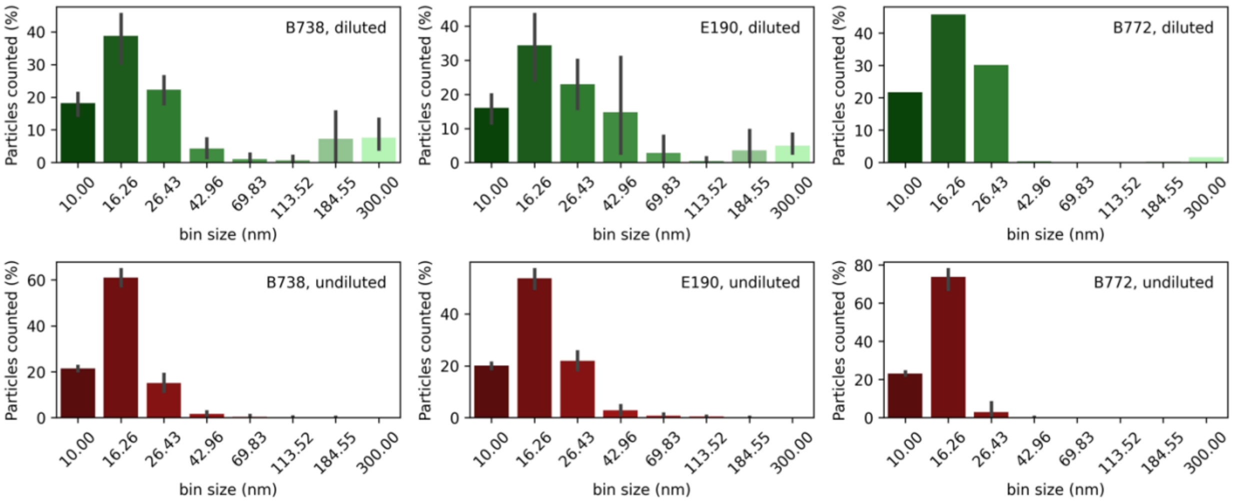

Figure S11: Average plume particle size distribution in the 8 Partector 2 Pro bins (in nm) for the B738 (left), E190 (center), B772 (right). Diluted and undiluted measurements depicted. Only arrival operations are included.

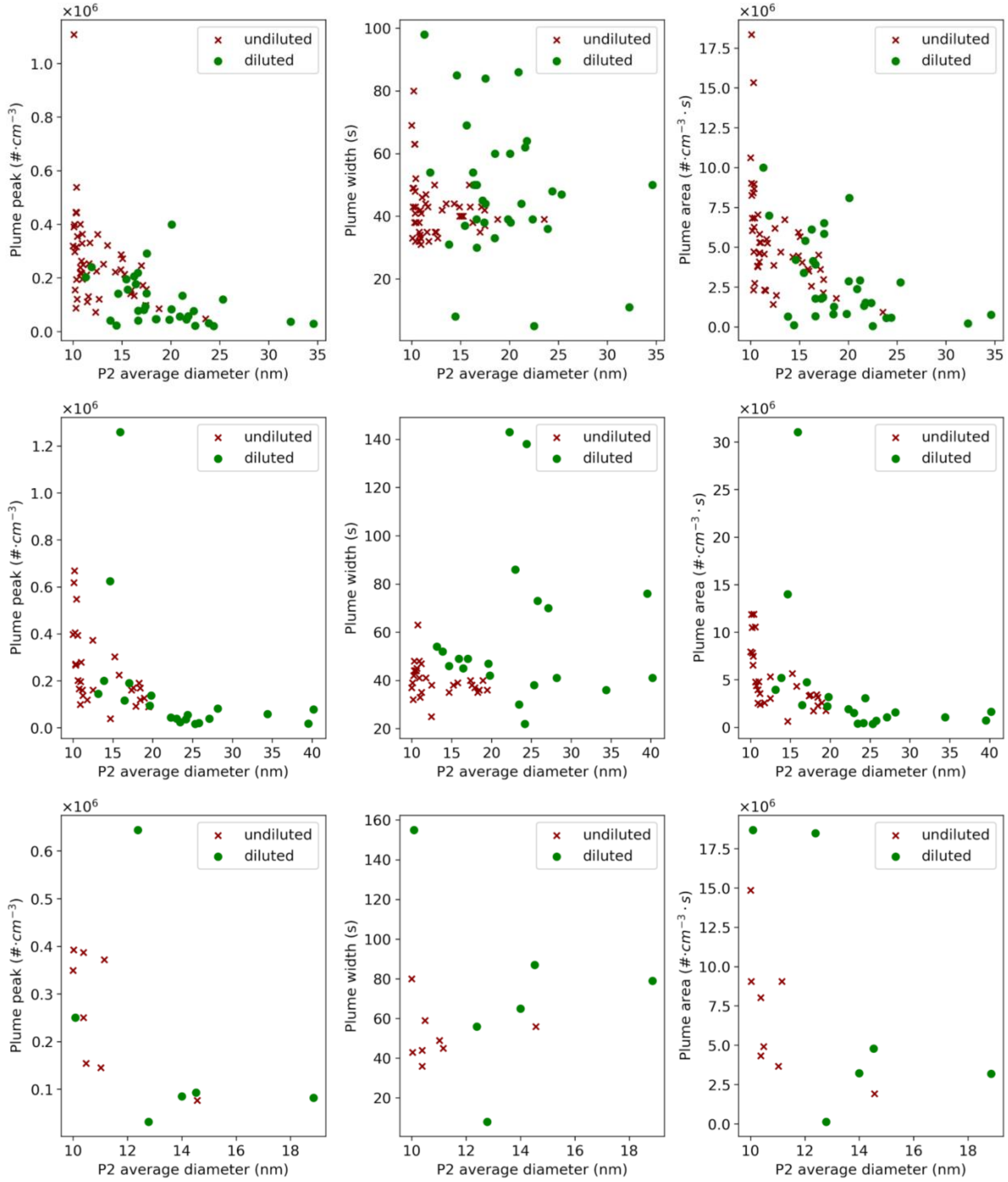

Figure S12: Average particle diameter (in nm) against plume peak (left), width (middle) and plume area right), measured by the Partector 2 for the B738 (top), E190 (middle), B772 (bottom). Diluted and undiluted measurements depicted. Only arrival operations are included.

S5 Particle size distribution for individual aircraft types

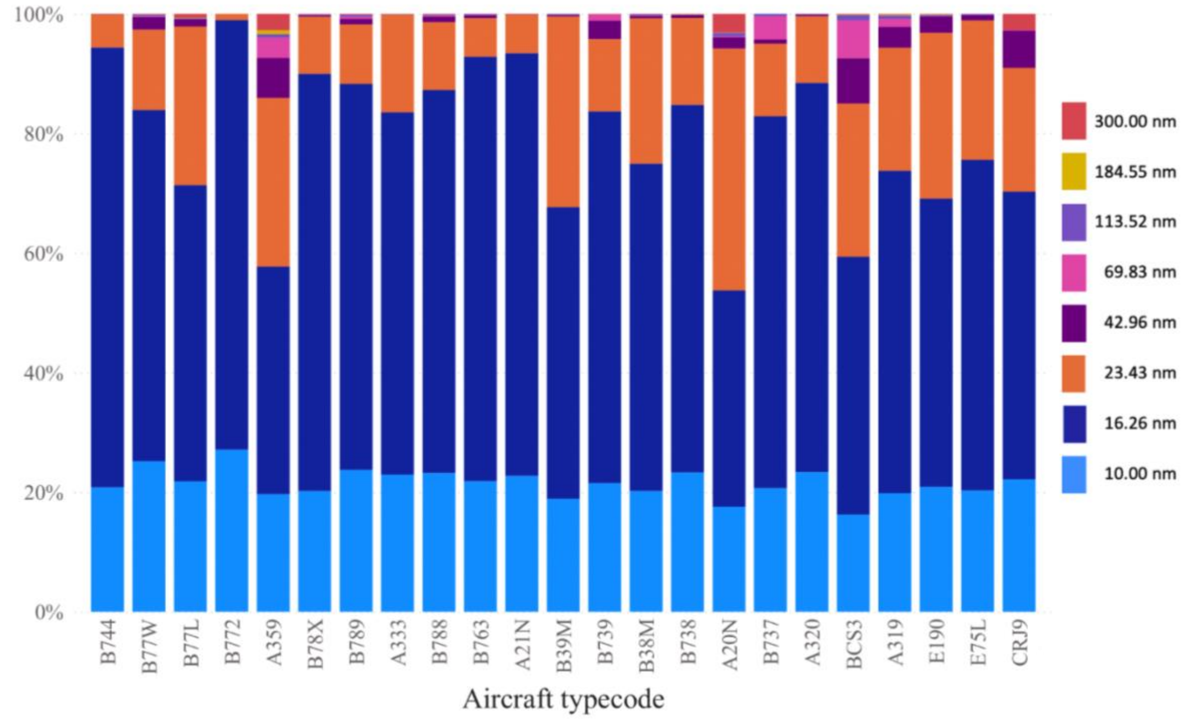

Figure S13: Percentage of particle number concentrations (#·cm^{-3}) that fall within each of the 8 size distribution channels between 10-300 nm of the Partector 2 Pro. The median percentage is shown for each aircraft type. Aircraft types are listed in decreasing Maximum Take-Off Weight (MTWO), from larger (left) to smaller (right) aircraft. Only arrival operations are included.

References

(1) Fissan, H.; Neumann, S.; Trampe, A.; Pui, D. Y. H.; Shin, W. G. Rationale and Principle of an Instrument Measuring Lung Deposited Nanoparticle Surface Area. *J Nanopart Res* **2007**, *9* (1), 53–59. https://doi.org/10.1007/s11051-006-9156-8.

(2) Fierz, M.; Meier, D.; Steigmeier, P.; Burtscher, H. Aerosol Measurement by Induced Currents. *Aerosol Science and Technology* **2014**, *48* (4), 350–357. https://doi.org/10.1080/02786826.2013.875981.

(3) Fierz, M.; Meier, D.; Steigmeier, P.; Burtscher, H. Miniature Nanoparticle Sensors for Exposure Measurement and TEM Sampling. *J. Phys.: Conf. Ser.* **2015**, *617* (1), 012034. https://doi.org/10.1088/1742-6596/617/1/012034.

(4) Asbach, C.; Todea, A. M.; Kaminski, H. Evaluation of a Partector Pro for Atmospheric Particle Number Size Distribution and Number Concentration Measurements at an Urban Background Site. *Aerosol Research* **2024**, *2* (1), 1–12. https://doi.org/10.5194/ar-2-1-2024.

(5) Hämeri, K.; Koponen, I. K.; Aalto, P. P.; Kulmala, M. The Particle Detection Efficiency of the TSI-3007 Condensation Particle Counter. *Journal of Aerosol Science* **2002**, *33* (10), 1463–1469. https://doi.org/10.1016/S0021-8502(02)00090-3.

(6) Bezantakos, S.; Biskos, G. Temperature and Pressure Effects on the Performance of the Portable TSI 3007 Condensation Particle Counter: Implications on Ground and Aerial Observations. *Journal of Aerosol Science* **2022**, *159*, 105877. https://doi.org/10.1016/j.jaerosci.2021.105877.

(7) Bezantakos, S.; Costi, M.; Barmpounis, K.; Antoniou, P.; Vouterakos, P.; Keleshis, C.; Sciare, J.; Biskos, G. Qualification of the Alphasense Optical Particle Counter for Inline Air Quality Monitoring. *Aerosol Science and Technology* **2021**.

(8) Barrett, S. R. H.; Britter, R. E.; Waitz, I. A. Impact of Aircraft Plume Dynamics on Airport Local Air Quality. *Atmospheric Environment* **2013**, *74*, 247–258. https://doi.org/10.1016/j.atmosenv.2013.03.061.
